# A role for the protease MALT1 in EBV-mediated B-cell transformation by Latent Membrane Proteins 1 and 2

**DOI:** 10.1038/s41375-026-03109-8

**Published:** 2026-08-13

**Authors:** Mélanie Juilland, Laurence Romy, Harun-Or Rashid, Sara Escudeiro Lopes, Silvia Podavini, David Vallois, Manhui Luo, Montserrat Gonzalez, Katerina Pandeva, Frédéric Frête, Chantal Décaillet, Laurence de Leval, Sylvia Rothenberger, Margot Thome

**Affiliations:** 1 Department of Immunobiology, University of Lausanne, Epalinges, Switzerland; 2 Institute of Pathology, Department of Laboratory Medicine and Pathology, Lausanne University Hospital and University of Lausanne, Lausanne, Switzerland; 3 Institute of Microbiology (IMU), Lausanne University Hospital and University of Lausanne, Lausanne, Switzerland

**Keywords:** Cell signalling, Tumour virus infections, Oncogenes

## Abstract

Epstein-Barr virus (EBV) is a human herpesvirus that causes a variety of hematological malignancies, in particular B-, T- and NK-cell lymphomas. Latency proteins of the virus contribute to lymphomagenesis by activating the transcription factor NF-κB, however, the molecular mechanism underlying EBV-driven NF-κB activation remains incompletely understood. Here we show that EBV latent membrane proteins LMP1 and LMP2A or LMP2B synergize to activate the protease MALT1, a key driver of NF-κB-mediated lymphocyte proliferation. EBV-positive B-cell lines co-expressing LMP1 and LMP2 proteins exhibited constitutive MALT1 activity, and treatment with a MALT1 inhibitor or silencing of latent membrane protein-1 (LMP1) or LMP2 impaired MALT1-dependent substrate cleavage. LMP1 physically interacted with the MALT1-binding partner BCL10, while LMP2A and LMP2B interacted with MALT1. LMP1 also colocalized with LMP2 in dotted structures, suggesting that LMP1 and LMP2 synergize via the coordinated formation of LMP-BCL10-MALT1 (LBM) complexes. Finally, MALT1 protease inhibition led to reduced expression of a set of NF-κB-driven genes, and reduced tumor growth in an EBV-positive lymphoma xenograft model. These findings establish a key role for the protease MALT1 in LMP1/2-mediated, NF-κB-driven cellular transformation and provide a rationale for inhibiting MALT1 to treat EBV-positive B-cell lymphomas that co-express LMP1 and LMP2.

**Figure:**
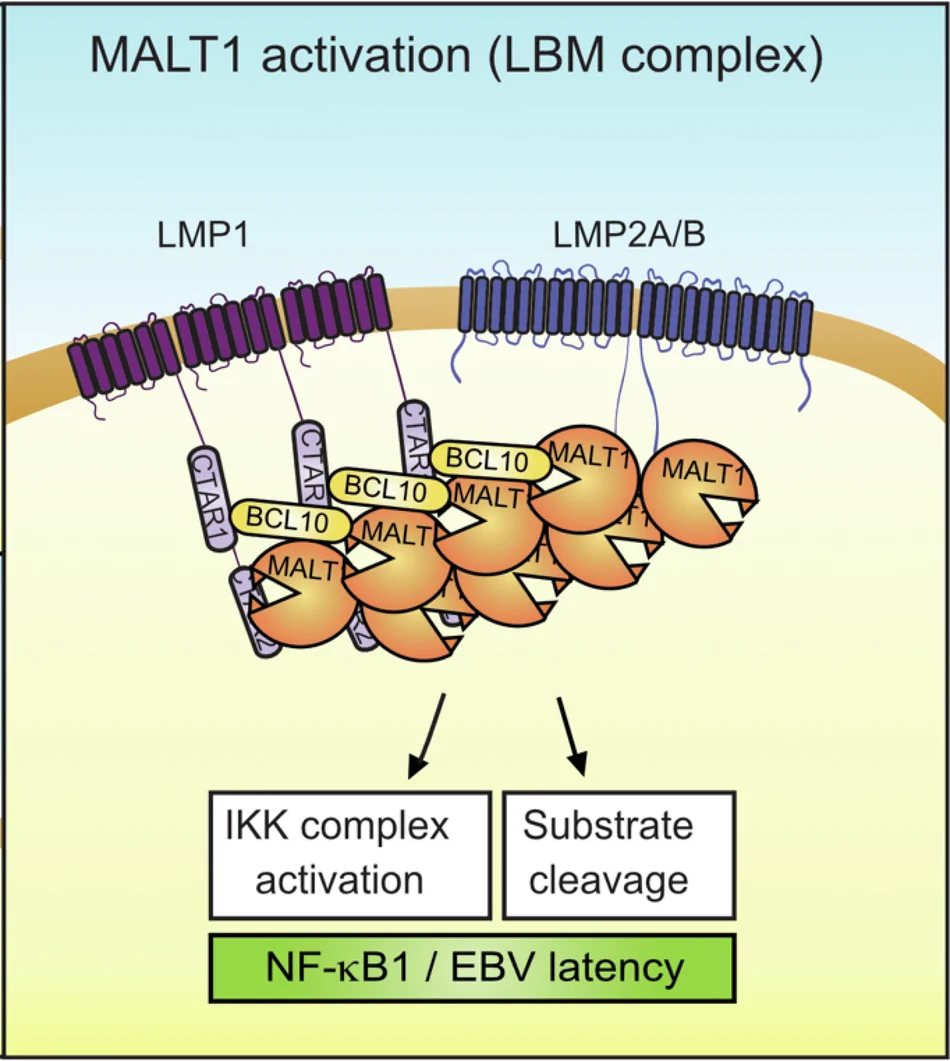

## Introduction

Epstein-Barr virus (EBV, also known as human herpesvirus-4, HHV-4) is a gamma herpes virus that is present in over 95% of the human population, infecting primarily B cells and epithelial cells [1]. In most individuals, EBV is asymptomatic, but in some, it can cause hematological malignancies or rare carcinomas, especially under immunosuppression [2]. EBV-related malignancies include Burkitt lymphomas (BL), classic Hodgkin lymphomas (HL) and diffuse large B cell lymphomas (DLBCL), as well as T cell lymphomas or NK-cell lymphomas, primary effusion lymphoma (PEL) and posttransplant lymphoproliferative disease (PTLD) [2, 3]. By promoting these malignancies, EBV is responsible for 2% of global cancer deaths [4, 5].

EBV, like other herpesviruses, has a latent and lytic life cycle, with different gene sets expressed in each phase. Cellular transformation requires the expression of latent genes [1, 2] encoding 6 EBV nuclear antigens (EBNA1, -2, -3A, -3B, 3 C, -LP), three latent membrane proteins (LMP1, LMP2A/B), two EBV-encoded small RNAs (EBER1/2), and BamH1 fragment A rightward transcripts (BARTs) [1]. These gene products give rise to four latency programs: 0, I, II, and III that express no (program 0) or different sets of latent proteins and are linked to specific EBV-related diseases [1, 2]. Program I (expressing EBNA1 and EBER) is associated with Burkitt lymphoma, program II (expressing EBNA1/2, LMP1 and LMP2A/B) with Hodgkin lymphoma, DLBCL, T-/NK-cell lymphomas, and nasopharyngeal carcinoma, and program III (expressing all EBNA proteins, LMP1 and LMP2A/B) with some DLBCL and lymphoblastoid cell lines (LCL), formed by EBV-mediated immortalization of B cells in vitro.

The EBV-encoded latent proteins contribute to lymphoid malignancies in various ways. A key oncogene essential for EBV-mediated B-cell transformation is the transmembrane protein LMP1, which shares signaling properties with CD40, a B-cell activating surface receptor [6]. LMP1 activity is ligand-independent, relying on dimerization and oligomerization [7]. LMP1 contains a cytosolic N-terminal segment, six transmembrane domains, and a cytosolic C-terminal segment with two important signaling motifs: CTAR1 and CTAR2 [8]. These motifs recruit signaling proteins that activate transcription factors of the NF-κB family via the canonical (NF-κB1) and non-canonical (NF-κB2) pathways [8–10]. The CTAR1 motif (P_204_-X-Q_206_-X-T_208_) recruits ubiquitin ligases of the TNF receptor associated factor (TRAF) family [9], and predominantly activates NF-κB2 [11, 12]. The CTAR2 motif (Y_384_-Y_385_-D_386_), on the other hand, binds the adapter protein TRADD and the kinase RIPK1, which together with TRAF2 and TRAF6 mediate activation of NF-κB1 via the IκB kinase (IKK) complex [10, 13–15]. IKK phosphorylates the NF-κB1 inhibitor IκBα, triggering its degradation and NF-κB1 nuclear entry [16]. Thus, LMP1 orchestrates the recruitment and activation of adapter proteins, ubiquitin ligases and kinases to promote NF-κB signaling in EBV-infected cells [6].

Another key latency gene of EBV is *LMP2*, which encodes two protein isoforms, LMP2A and LMP2B, as a result of alternative transcription [17]. LMP2A has 12 transmembrane regions and cytoplasmic N- and C-terminal regions. The C-terminal region mediates homo- and heterodimerization of LMP2 protein isoforms [18, 19]. The N-terminal region includes multiple tyrosine phosphorylation sites, including an immunoreceptor tyrosine-based activation motif (ITAM), which recruit tyrosine kinases and mimic B-cell receptor (BCR) signaling [17]. Through PY motifs in its N-terminal tail, LMP2A additionally recruits ubiquitin ligases that promote its rapid turnover and that of BCR components; this process may prevent BCR-induced lytic reactivation and help EBV to remain latent [17]. LMP2B lacks the N-terminal signaling region, but through formation of heterodimers with LMP2A, it modulates its distribution and function [18–20]. The role of LMP2 proteins in EBV-induced B-cell transformation in vitro remains controversial and possibly limited to B cells with low or absent BCR expression [21–24]. However, transgenic expression of LMP2A in mice promotes B-cell survival in vivo [25–27] and accelerates c-Myc-driven lymphomagenesis [28]. Moreover, co-expression of LMP1 and LMP2A has a cooperative effect on c-Myc-driven B-cell proliferation in vivo [28].

The transcription factor NF-κB is crucial for lymphocyte proliferation and survival. Antigen receptor (AgR) activation triggers NF-κB through assembly of the oligomeric CBM complex, which includes the proteins CARMA1 (also known as CARD11), BCL10, and MALT1 [29]. CBM formation requires the AgR-induced, PKC-mediated phosphorylation of CARMA1 [30]. This induces a conformational change and oligomerization of CARMA1, which nucleates assembly of preformed MALT1-BCL10 dimers into polymeric fibers [31–33]. This assembly enables MALT1 to recruit TRAF6 and promote IKK-dependent NF-κB1 activation [32, 34–36]. As a consequence of CBM assembly, MALT1 also acquires protease activity and enhances NF-κB activation by cleaving negative regulators of the pathway such as RelB [37] and A20 [38].

Constitutive CBM formation and MALT1 activity are hallmarks of various hematological malignancies, including diffuse large B-cell lymphoma (DLBCL) of the activated B-cell (ABC) subtype and mantle cell lymphoma, which are driven by activating mutations in components of the AgR signaling pathway or constitutive engagement of self-reactive AgRs [39]. We have previously reported that MALT1 is activated by K13 and K15, latent proteins of the EBV-related Kaposi’s sarcoma-associated herpesvirus (KSHV, also known as human herpesvirus-8, HHV-8), and that MALT1 inhibitors reduce growth of KSHV-induced primary effusion lymphomas in xenografts [40]. Here, we show that MALT1 was constitutively activated in an LMP1- and LMP2-coexpressing subset of EBV^+^ B-cell lymphoma models and EBV-transformed lymphoblastoid cell lines, and that LMP1 and LMP2A/B have an essential role in MALT1 activation. Furthermore, MALT1 inhibitors delayed the growth of EBV^+^ DLBCL in a xenograft model. Thus, MALT1 inhibitors, currently in clinical trials [41], may offer a treatment option for an LMP1^+^LMP2^+^ subset of EBV-driven malignancies.

## Materials and methods

### Cell culture, cell treatment and reagents

293T cells (a kind gift of the late Jürg Tschopp, UNIL, Lausanne, Switzerland) were cultured in DMEM (Life Technology/ThermoFischer, Reinach, Switzerland) supplemented with 10% FCS (Biowest**/**VWR, Nyon, Switzerland) and antibiotics (Pen/Strep, Life technology/ThermoFischer, Reinach, Switzerland). The human GCB DLBCL cell line BJAB (a kind gift of Pascal Schneider, UNIL, Lausanne, Switzerland) and its transduced derivatives were cultured in RPMI 1640 (Life Technology/ThermoFischer, Reinach, Switzerland) supplemented with 10% FCS. The human ABC DLBCL cell lines, OCI-Ly3, HBL1, U2932 and TMD8 (a kind gift of Louis Staudt, NCI, Bethesda, US) were cultured in RPMI 1640 supplemented with 20% FCS. EBV-positive cell lines Farage and VAL (DLBCL), VL51 (SMZL), Jijoye, RAJI, Namalwa, BL41 and Ramos (Burkitt lymphoma), the lymphoblastoid cell lines (LCL) ADW, ADZ, HEL, NDW, POO, KDW, WWT, WWT1-3 and CB33, and B95.8 (a marmoset B-lymphoblastoid cell line), were cultured in RPMI 1640 supplemented with 10% FCS. EBV-positive cell lines were kindly provided by Francesco Bertoni (USI, Bellinzona, Switzerland) and Elisa Oricchio, EPFL, Lausanne, Switzerland), and LCL were kindly provided by Hermann Eibel, University of Freiburg, Germany. All lines were tested for mycoplasma and validated by STR profiling (Microsynth, Balgach, Switzerland). MLT-748 (MedChemExpress, Sollentuna, Sweden), z-LVSR-fmk (Bachem, Bubendorf, Switzerland), Ibrutinib (Selleckchem/Ambeed, Buffalo Grove, IL, US) and Gö6983 (Sigma-Aldrich/Merck, Buchs, Switzerland) were diluted in DMSO and used to treat cells at a final concentration of 3.125 nM for Ibrutinib, 12.5 nM for Gö6983, 10 μM for MLT-748 and 10 μM for z-LVSR-fmk.

### Antibodies

Antibodies used included affinity-purified rabbit anti-LMP2A/B (prepared in-house, generated against a C-terminal peptide: LESEERPPTPYRNTV), anti-EBV LMP1 (clone S12, Merck, Buchs, Switzerland), anti-BCL10 (331.3, Santa Cruz Biotechnology, Heidelberg, Germany), affinity purified rabbit anti-cleaved BCL10 [42], anti-FLAG (M2) and polyclonal anti-VSV (both from Merck, Buchs, Switzerland), monoclonal anti-VSV (P5D4, Labforce AG, Muttenz, Switzerland), anti-CARMA1 (1D12), anti-CYLD (D1A10) and anti-RelB (4954) (all three from Cell Signaling Technology/Bioconcept, Allschwill, Switzerland), anti-Tubulin (B-5-1-2, Merck, Buchs, Switzerland), anti-β-Actin (Santa Cruz Biotechnology, Heidelberg, Germany), anti-Strep-HRP (IBA BioTAGnology/Lucerna-Chem AG, Lucern, Switzerland) and anti-GFP (Enzo LifeSciences, Lausen, Switzerland). Affinity-purified MALT1 antibodies have been previously described [42]. For microscopy, we used anti-MALT1 (sc-46677) and anti-LMP2 (sc-101315) from Santa Cruz Biotechnology, Heidelberg, Germany. Secondary antibodies included horseradish peroxidase–coupled goat anti-mouse (115-035-146) and anti-rabbit (111-035-144) from Jackson ImmunoResearch (Ely, Cambridgeshire, UK).

### Transfection and transduction of cells, gene silencing

Transient transfection of 293T cells and lentiviral transduction of cell lines were performed as previously described [42, 43]. For inducible gene silencing, cells were transduced with a lentiviral vector (pLKO-Tet-On, Addgene, Watertown, MA, US) containing shRNA sequences specific for LMP1 (CCAGTTCAGCTAAGCTACTAT), LMP2A and LMP2B (TGGCGGACTAC AAGGCATTTA and TTGTCACTTTCTTTGCAATTT), MALT1 (GTCACAGAATTGAGTGATTTC), CARMA1 (GCTGGTCAACAGGATCTACAA) or a scrambled sequence (CTAAGGTTAAGTCGCCCTCG). Expression of shRNA was induced using 2 μg/mL doxycycline for 72 h (Applichem/Axonlab, Le Mont-sur-Lausanne, Switzerland).

### Cell lysis and immunoblot analysis

Cells were lysed in lysis buffer containing 50 mM HEPES, pH 7.5, 150 mM NaCl, 1% Triton X-100, protease inhibitors (Complete, Roche Diagnostics, Rotkreuz, Switzerland) and phosphatase inhibitors NaF, Na_4_P_2_O_7_ and Na_3_VO_4_ (Merck, Buchs, Switzerland). Samples were boiled in reducing SDS sample buffer, separated by SDS polyacrylamide gel electrophoresis (SDS-PAGE) and analyzed by immunoblot as described [42].

### Statistical analysis

Parametric two-tailed Student’s *t* test or one-way ANOVA with Dunnett correction were used for statistical analysis; *P* ≤ 0.05 were considered statistically significant.

## Results

### MALT1 is constitutively active in a subset of EBV-transformed B-cell lines

**Fig. 1:**
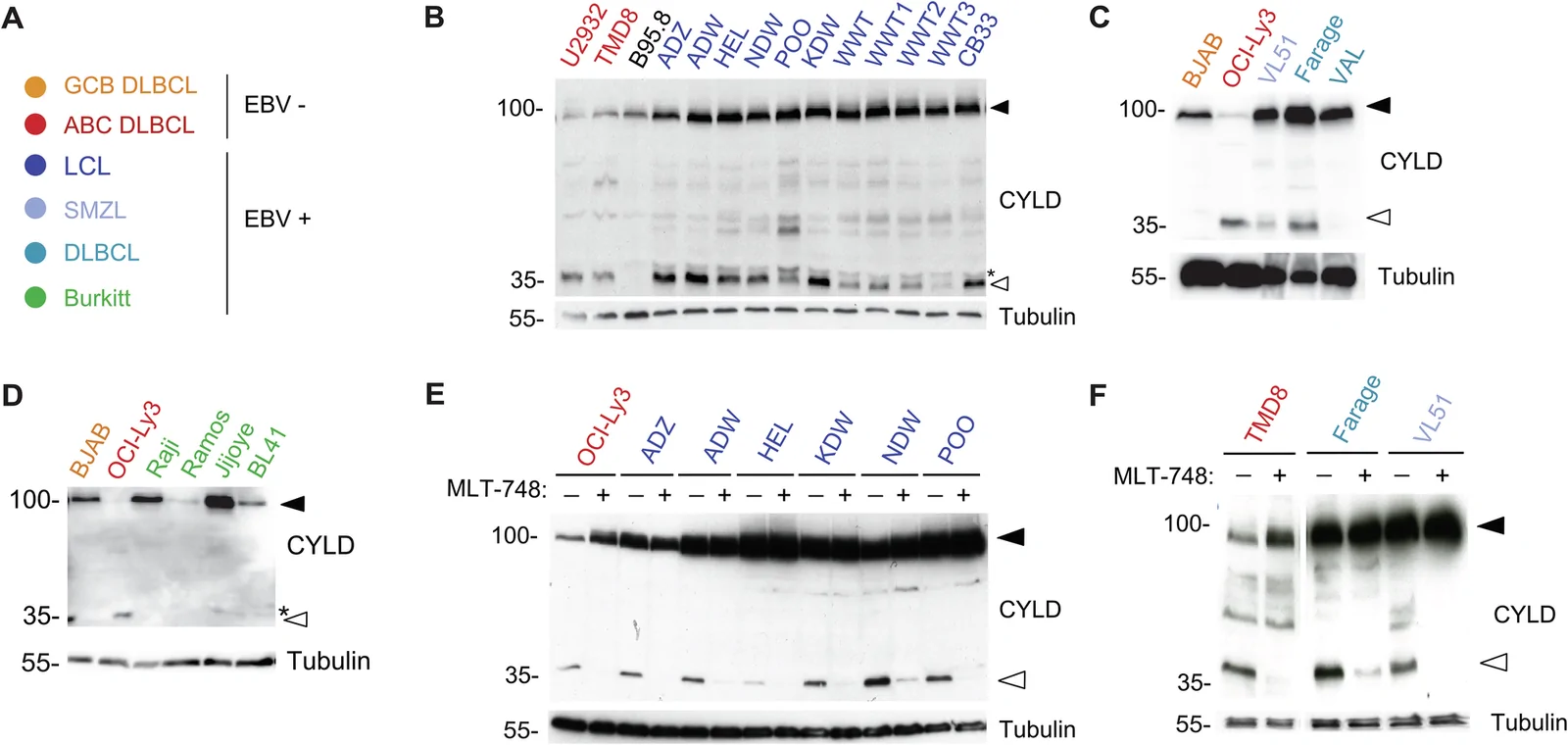
MALT1 is constitutively active in a subset of EBV-positive lymphoma cell lines. **A** Color code for the different EBV-negative (orange: GCB DLBCL, red: ABC DLBCL) and EBV-positive cell lines (dark blue: LCL, pale violet: SMZL, turquoise blue: EBV^+^ DLBCL, green: Burkitt lymphoma). **B**–**F** Immunoblot for CYLD was used to monitor MALT1 activity in lysates of ABC DLBCL (OCI-Ly3, U2932 and TMD8), GCB DLBCL (BJAB), B98.8 (an EBV-producing marmoset B-lymphoblastoid cell line), EBV-positive LCL (ADZ, ADW, HEL, NDW, POO, KDW, WWT, WWT1-3, CB33), EBV-positive SMZL (VL51) and EBV-positive Burkitt lymphoma (Raji, Ramos, Jijoye and BL41) cell lines. Black and open arrowheads indicate noncleaved and cleaved forms of CYLD, respectively. **E**, **F** Indicated cell lines were incubated for 24 h with or without the MALT1 inhibitor MLT-748. **B**–**F** Tubulin served as loading control throughout, and an asterisk indicates a non-specific band. Positions of molecular weight markers are indicated in kDa. Data are representative of three independent experiments.

Constitutive NF-κB activation is a characteristic of lymphoid tumors induced by oncogenic viruses such as EBV or KSHV. We recently showed that the KSHV latent proteins K13 and K15 activate NF-κB through the protease MALT1 [40]. Since K15 is homologous to EBV’s LMP2A, we hypothesized a similar mechanism in EBV-induced B-cell transformation. Therefore, we screened for MALT1 activity in various EBV^-^ and EBV^+^ B-cell lines (see color code used throughout this study, depicted in Fig. 1A), by assessing the cleavage status of the MALT1 substrate CYLD [44]. As positive controls, we used OCI-Ly3, U2932 or TMD8 cell lines, which are derived from the ABC subtype of DLBCL (ABC DLBCL, labeled in red), characterized by constitutive BCR signaling and MALT1 activity, and as a negative control, we used BJAB, a cell line derived from the germinal center B cell (GCB) subtype of DLBCL (GCB DLBCL, labeled in orange), in which MALT1 is inactive [45, 46]. MALT1 activity was detectable in multiple lymphoblastoid cell lines (LCL) derived from EBV-transduced primary human B cells (labeled in dark blue), but not in the marmoset LCL B95.8 cell line that is used for EBV virus production (Fig. 1B). MALT1 activity was also detectable in one out of two EBV^+^ DLBCL cell lines (Farage, but not VAL, labelled in turquoise blue), and in the splenic marginal zone lymphoma (SMZL) cell line VL51 (labelled in pale violet) (Fig. 1C), despite consistently observing that higher proportions of CYLD were cleaved in the ABC DLBCL models used as a positive control [45, 46] (Fig. 1B, C). Whilst CYLD cleavage was most easily picked up, cleavage of other MALT1 substrates such as BCL10 and RelB were also detectable in the lymphoblastoid cell line KDW and in VL51 cells (Supplementary Fig. 1A). In contrast, MALT1 activity was absent in EBV^+^ Burkitt lymphoma models (Raji, Ramos, Jijoye, BL41 and Namalwa, labeled in green), and the GCB DLBCL cell line BJAB, used as negative control [45, 46] (Fig. 1D). Substrate cleavage was MALT1-dependent, as it could be inhibited with the allosteric MALT1 inhibitor MLT-748 (ref. [47]) (Fig. 1E, F, Supplementary Fig. 1A) or the competitive MALT1 inhibitor z-LVSR [48] (Supplementary Fig. 1B and 1C), or by inducible silencing of MALT1 expression (Supplementary Fig. 1D). Thus, MALT1 is constitutively active in a subset of EBV^+^ B-cell lines.

### Constitutive MALT1 activation in EBV^+^ cells is independent of the BCR-proximal signaling pathway

**Fig. 2:**
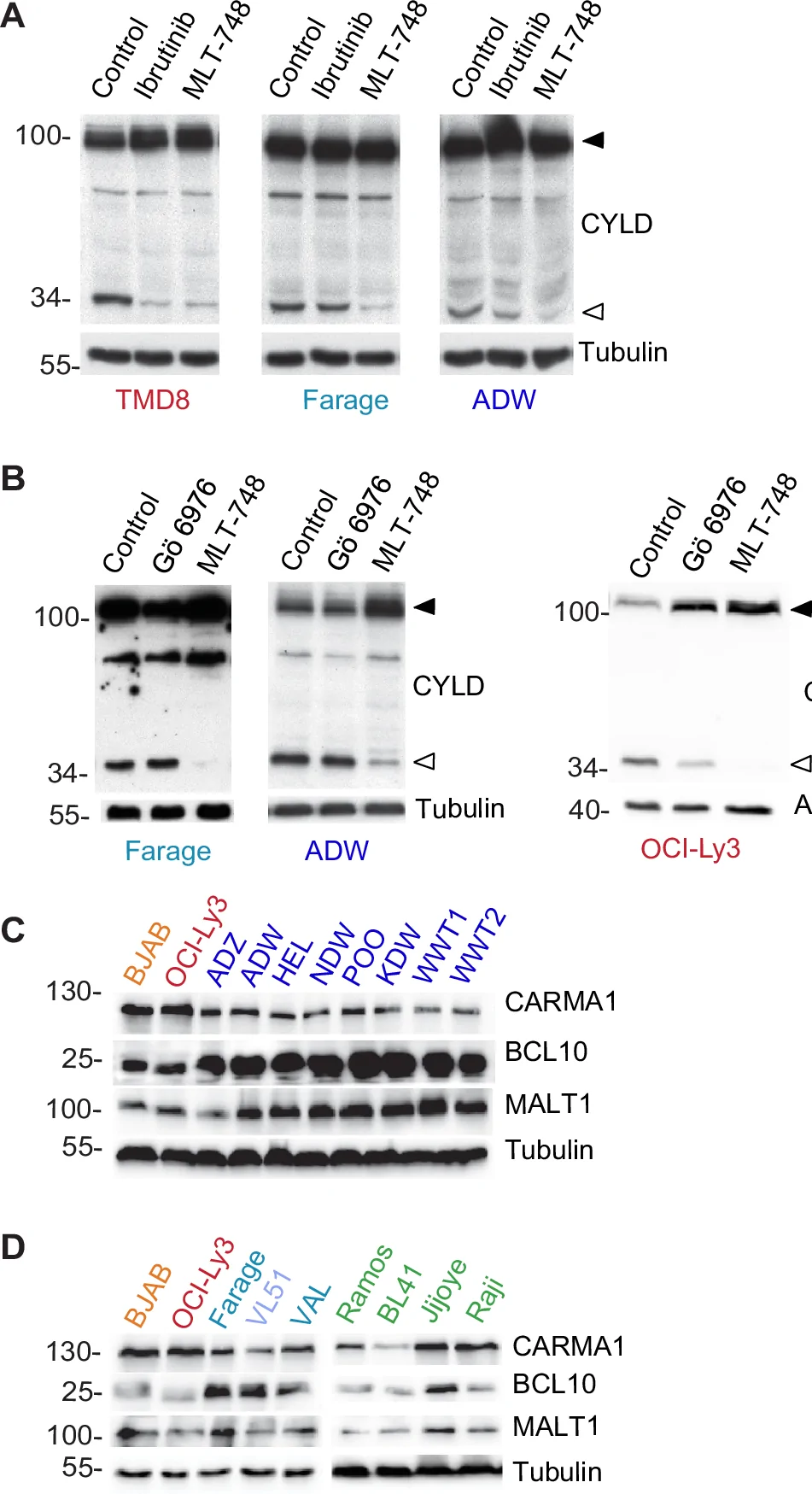
Btk and PKC activities are not required for MALT1 activation in EBV-positive lymphoma cell lines. **A**, **B** Immunoblot analysis of CYLD cleavage as a readout of MALT1 activity in lysates of ABC DLBCL (TMD8, OCI-Ly3), DLBCL (Farage) and LCL (ADW) cells treated with (**A**) the BTK inhibitor Ibrutinib, (**B**) the PKC inhibitor Gö6976 or (A-B) the MALT1 inhibitor MLT-748. **C** Immunoblot analysis of CARMA1, MALT1 and Bcl10 expression in lysates of ABC DLBCL (OCI-Ly3), GCB DLBCL (BJAB), EBV^+^ DLBCL (VAL, Farage), LCL (ADZ, ADW, HEL, NDW, POO, KDW, WWT1, WWT2), SMZL (VL51) and Burkitt lymphoma (Ramos, BL41, Raji, Jijoye) cell lines. **A**–**D** Tubulin served as loading control throughout. Black and open arrowheads indicate noncleaved and cleaved forms of CYLD, respectively. Positions of molecular weight markers are indicated in kDa. Data in all panels are representative of two independent experiments.

To explore molecular requirements of MALT1 protease activation in specific EBV^+^ cell lines, we examined whether BCR-proximal signaling components such as BTK, PKCβ and its substrate CARMA1 were involved, in light of their known requirement for BCR-induced MALT1 activation. Using the BTK inhibitor Ibrutinib or Gö6976, an inhibitor of PKCα, PKCβ, and PKCγ, we found little or no effect on MALT1 activity in EBV^+^ cell lines with constitutive MALT1 activity (Farage and ADW), whilst Ibrutinib and Gö6976 inhibited MALT1 activity in the ABC DLBCL cell line OCI-Ly3, as expected [49] (Fig. 2A, B). All three cell lines responded to the MALT1 inhibitor MLT-748, which efficiently inhibited CYLD cleavage (Fig. 2A, B). Finally, we assessed the effect of inducible CARMA1 silencing, which in contrast to MALT1 silencing had no effect on CYLD cleavage (Supplementary Fig. 1D–E).

Subsequently, we selected a panel of 54 genes that are frequently mutated in B-cell lymphomas and used for diagnostic purposes, to assess potential presence of gain-of-function mutations in the EBV^+^ cell lines with constitutive MALT1 activity (Supplementary Table 1) [50]. These include genes with roles in BCR-proximal signaling and formation of the CBM complex, such as CD79A/B and CARMA1, which are recurrently mutated in lymphomas [51, 52]. Genomic DNA from Farage, VL51, and CB33 cells was sequenced using Hybrid Capture-Based Next Generation Sequencing. No gain-of-function mutations in CD79, CARMA1, or other CBM complex regulators were found in these cells (Supplementary Table 1).

Finally, we assessed for potential alterations in the expression and/or modification (e.g., altered migration pattern) of CBM components by Western blot. No consistent differences in expression or modification of MALT1, CARMA1, or BCL10 were observed between cell lines with MALT1 activity, including OCI-Ly3, LCL lines, Farage, VL51, and EBV^+^ cell lines that lack MALT1 activity (VAL, Raji and Jijoye) (Fig. 2C, D).

Thus, a subset of EBV^+^ B-cell lymphoma lines show constitutive MALT1 activity without evidence for chronic BCR-induced signaling.

### MALT1 activity is driven by EBV latent membrane proteins

**Fig. 3:**
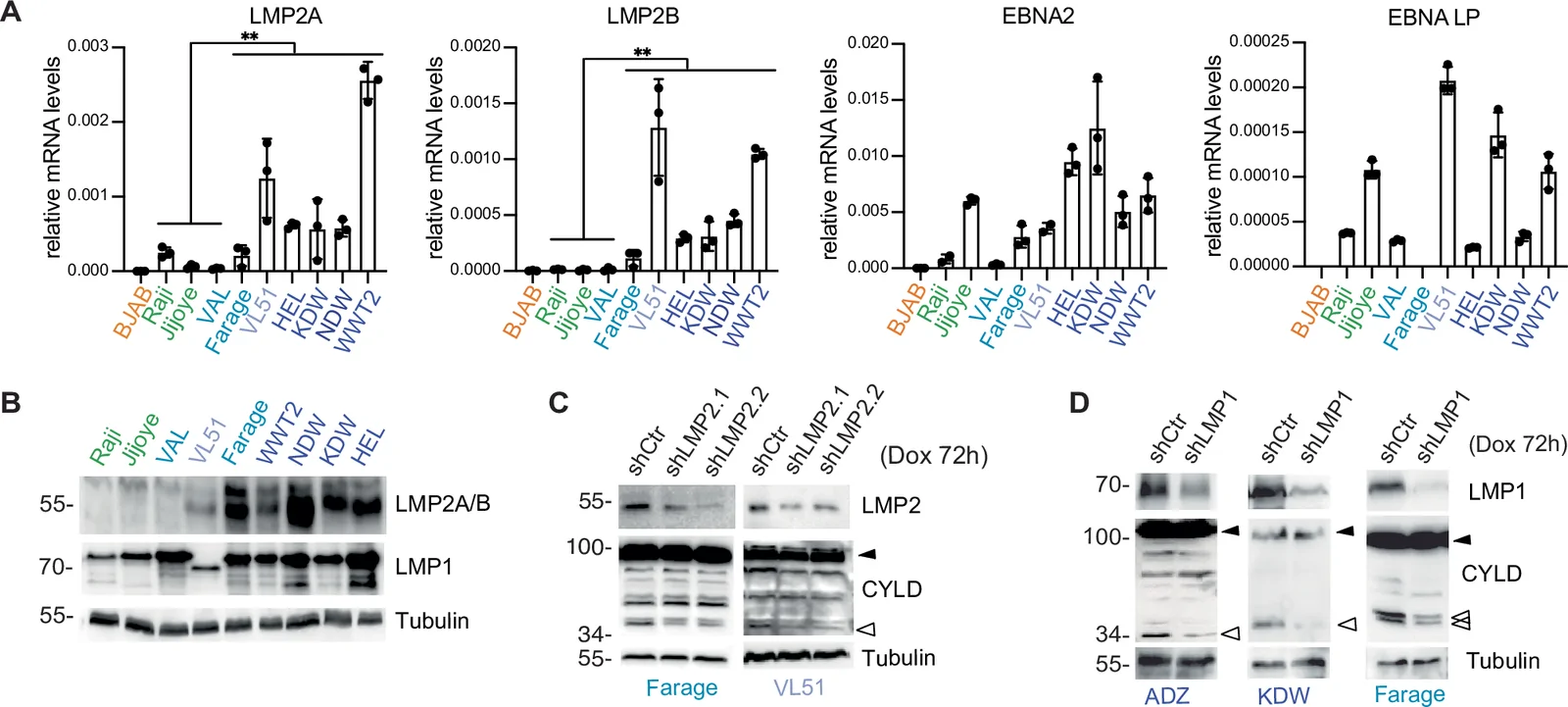
LMP proteins promote MALT1 activation. **A** RT-PCR analysis of the expression of indicated viral latent genes in GCB DLBCL (BJAB), EBV^+^ DLBCL (VAL, Farage), LCL (HEL, NDW, KDW, WWT2), SMZL (VL51) and Burkitt lymphoma (Raji, Jijoye) cell lines. Relative mRNA levels (normalized to GAPDH) are shown. **B** Immunoblot analysis of LMP2A/B and LMP1 expression in lysates of EBV^+^ DLBCL (VAL, Farage), LCL (HEL, NDW, KDW, WWT2), SMZL (VL51) and Burkitt lymphoma (Raji, Jijoye) cell lines. **C** EBV^+^ GCB DLBCL cell line (Farage) and SMZL VL51 were transduced with two different Dox-inducible shRNAs for LMP2 or a scrambled control shRNA and analyzed 3 days after induction by immunoblot, as indicated. CYLD was used to monitor MALT1 activity. **D** LCL (ADZ, KDW) or an EBV^+^ DLBCL cell line (Farage) were transduced with indicated, Dox-inducible shRNAs and analyzed 3 days after induction by immunoblot, as indicated. CYLD was used to monitor MALT1 activity. **B**–**D** Tubulin served as loading control throughout. Black and open arrowheads indicate noncleaved and cleaved forms of CYLD, respectively. Positions of molecular weight markers are indicated in kDa. Data in all panels are representative of two independent experiments.

Having excluded a role for altered BCR signaling, we examined the possibility that latent proteins associated with EBV malignancies could drive MALT1 activation. EBV-infected cells express four latency programs linked to different forms of EBV-driven malignancies [1]. The MALT1-active cell lines identified here should express latency programs II or III, while inactive cell lines (mainly Burkitt lymphomas) are known to express latency program I. We monitored viral latent gene expression and proteins in cell lines that are EBV^-^ (BJAB) or EBV^+^ (all others) by RT-PCR and western blot. Within the group of EBV^+^ cell lines, higher mRNA expression of LMP2A and LMP2B was found in MALT1-active cell lines (Farage, VL51 and LCLs) compared to MALT1-inactive cell lines (VAL and Burkitt’s lymphoma), while no such correlation was observed for other latent genes such as EBNA2 or EBNA LP (Fig. 3A). Analysis of protein levels showed that co-expression of LMP1 with LMP2A/B correlated with MALT1 activity in VL51, Farage, and LCL lines (Fig. 3B, compare with Fig. 1B, C and F). Comparison of the two EBV-positive DLBCL cell lines Farage and VAL revealed that Farage, but not VAL cells expressed LMP2, which correlates with MALT1 activity detectable in Farage but not VAL cells (Fig. 1C). Consistent with an important role for LMP2 expression in MALT1 activation, inducible silencing of LMP2A/B reduced MALT1 activity, monitored by assessing CYLD cleavage, in the EBV^+^ DLBCL cell line Farage and the SMZL cell line VL51 (Fig. 3C).

Finally, we assessed whether the major EBV latent protein, LMP1, is required for MALT1 activity. No significant differences in LMP1 expression were found between MALT1-active and inactive cell lines, all of which expressed LMP1 (Fig. 3D). However, inducible silencing of LMP1 in the EBV^+^ DLBCL cell line Farage and in the LCL lines ADZ and KDW led to a clear decrease in MALT1 activity, as evidenced from reduced CYLD cleavage in LMP1-silenced cells (Fig. 3D). Thus, co-expression of LMP1 and LMP2A/B is likely necessary for MALT1 activation and could explain why MALT1 is active only in a subset of EBV^+^ B-cell lines corresponding to latency stages II and III.

### LMP1 and LMP2A/B synergistically activate MALT1 and NF-κB

**Fig. 4:**
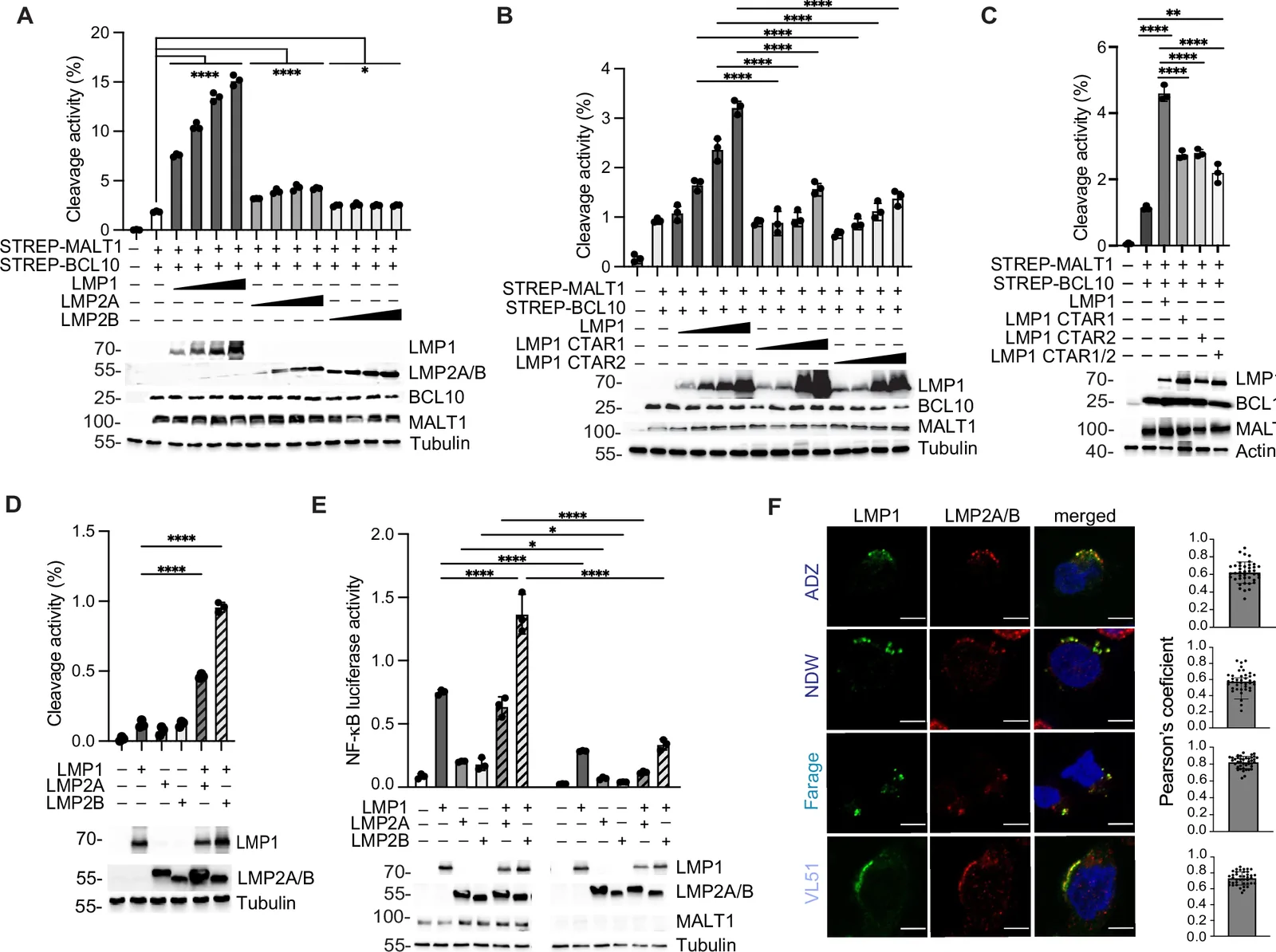
MALT1 and NF-κB activation are driven by EBV latent membrane proteins in a synergistic manner. **A**–**D** 293T cells were transfected with an eYFP-LVSR-eCFP MALT1 activity reporter plasmid and the indicated combinations of expression constructs for MALT1, BCL10, LMP1, LMP2A and LMP2B. LMP1 mutations of CTAR1, CTAR2 or both were additionally used in panels **B** and **C**. The proportion of cells with MALT1-dependent reporter cleavage was assessed by flow cytometry, and protein expression was assessed by western blot. **E** 293T cells stably transduced with silencing constructs for MALT1 (MALT1 KO) or a control vector were transfected with an NF-κB reporter construct, together with the indicated combinations of LMP1, LMP2A and LMP2B constructs. NF-κB activation was monitored by luciferase assay. Expression of transfected constructs and silencing efficiency was monitored by western blot. **F** LCL (ADZ, NDW), DLBCL (Farage) and SMZL (VL51) cell lines were stained for LMP1 (in green) and LMP2 (in red) and their endogenous colocalization (in yellow) was analyzed by fluorescence microscopy. Colocalization was quantified using Pearson’s coefficient. Bars indicate 5 μm. **A**–**E** Tubulin blot served as a loading control. Data in all panels are representative of two independent experiments.

To test whether individual LMP proteins alone can activate MALT1, we assessed their effects using a FRET-based MALT1 protease activity reporter in 293T cells [43]. Co-expression of low levels of MALT1 and BCL10 with increasing doses of LMPs revealed that LMP1 strongly activated MALT1, while LMP2A and LMP2B had weak effects (Fig. 4A). LMP1-induced MALT1 activation was significantly reduced by mutation of the CTAR1 or CTAR2 motifs of LMP1 (Fig. 4B), which are important for NF-κB2 and NF-κB1 activation, respectively [8–10]. Mutation of both, CTAR1 and CTAR2 motifs further reduced MALT1 activation (Fig. 4C). To determine if LMP1 could synergize with LMP2A or LMP2B, we then investigated the effect of their co-expression on the activation of endogenous MALT1, by titrating down LMP protein levels to have minimal MALT1 activation when expressed alone. Under these conditions, we observed a clear synergy between LMP1 and LMP2A or LMP2B (Fig. 4D). Unexpectedly, the synergy was even stronger with LMP2B, which lacks the N-terminal cytoplasmic tail present in LMP2A.

We subsequently tested whether LMP-mediated NF-κB transcription depended on MALT1, using an NF-κB luciferase reporter assay. Silencing of MALT1 strongly reduced NF-κB activation by all three LMP proteins in 293T cells (Fig. 4E), suggesting that LMP-dependent NF-κB activation requires MALT1. Furthermore, a MALT1-dependent synergy between LMP1 and LMP2B, but not between LMP1 and LMP2A, was observed in activating NF-κB (Fig. 4E). To explore the mechanism behind this synergy, we examined the subcellular localization of LMP1 and LMP2 proteins in LCL (ADZ, NDW), EBV^+^ DLBCL (Farage), and SMZL (VL51) cell lines using confocal microscopy. This revealed partial colocalization of LMP1 and LMP2 proteins in a perinuclear region (Fig. 4F). Thus, LMP1 and LMP2 proteins co-localize and activate MALT1, and consequently NF-κB, in a synergistic manner.

### LMP1 recruits BCL10, via its CTAR1 domain, and LMP2A recruits MALT1

**Fig. 5:**
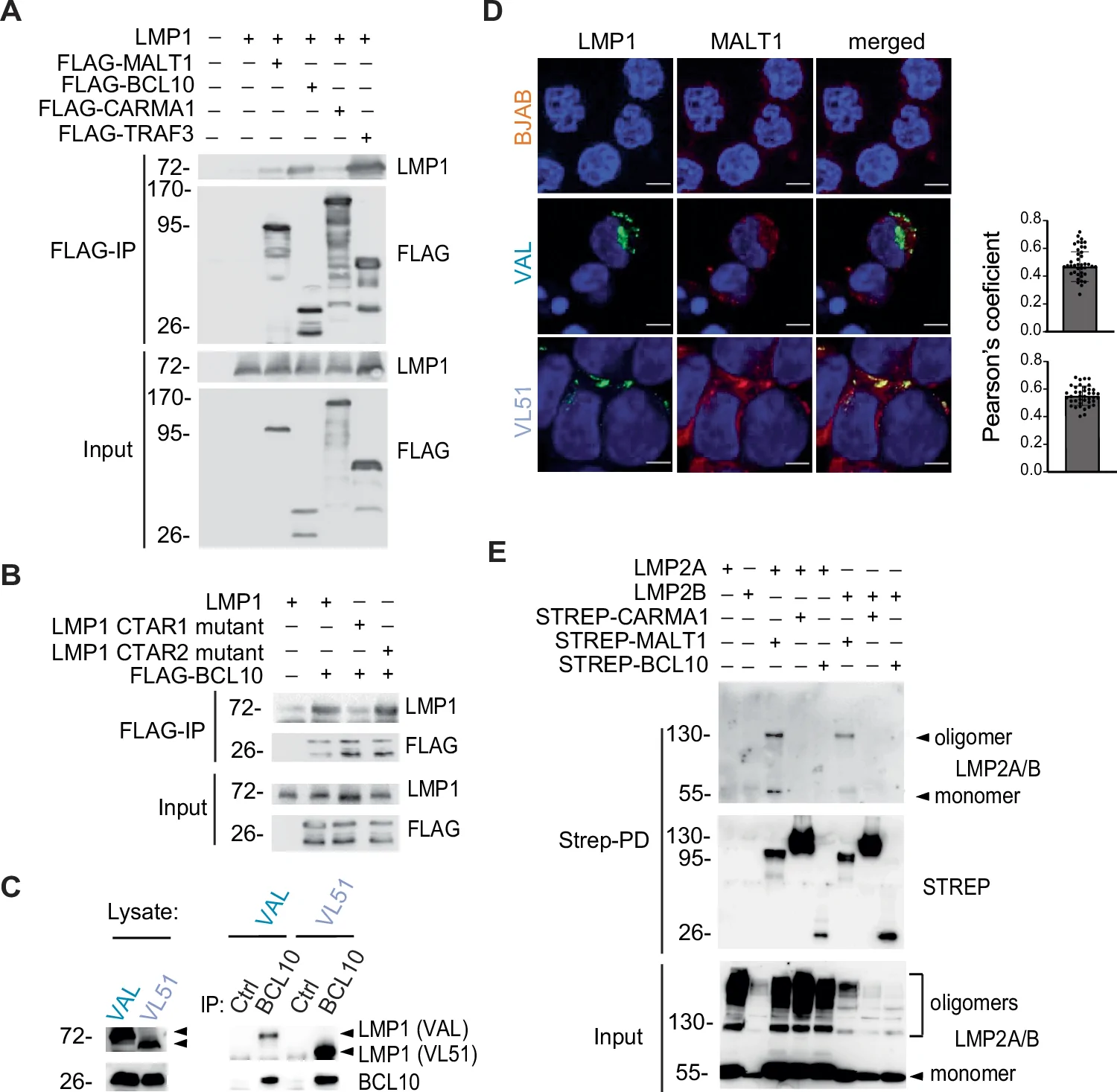
LMP1, LMP2A and LMP2B physically interact with CBM components. 293T cells were co-transfected with the indicated combinations of FLAG-tagged expression constructs for MALT1, BCL10 and CARMA1 together with (**A**) LMP1, or (**B**) LMP1 CTAR1 or CTAR2 mutants. Protein expression and binding of LMP1 constructs to precipitated CARMA1, MALT1 or BCL10 was monitored by western blot. **C** EBV^+^ cell lines without (VAL) or with constitutive MALT1 activity (VL51) were lysed, and proteins were precipitated using anti-BCL10 or a mouse control antibody (Ctrl). Proteins in lysates and co-immunoprecipitating proteins were analyzed by western blot using anti-LMP1 or anti-BCL10. Black arrowhead indicates the differently migrating LMP1 species present in the two cell lines. **D** VAL and VL51 cells were stained for endogenous LMP1 (green) and MALT1 (red) and their colocalization (yellow) was analyzed by fluorescence microscopy. Colocalization was quantified using the Pearson’s coefficient. BJAB cells were used as a negative control for LMP1 staining. Bars indicate 5 μm. **E** 293T cells were co-transfected with the indicated combinations of STREP-tagged expression constructs. Protein expression and binding of LMP2A and LMP2B to precipitated CARMA1, MALT1 or BCL10 was monitored by western blot. Data in all panels are representative of two independent experiments.

Based on our microscopy findings, we hypothesized that LMPs promote NF-κB and MALT1 activation by physically recruiting components of the CBM complex to a common location. We, therefore, tested whether LMP1 interacts with CARMA1, MALT1, or BCL10 in 293T cells, using the known LMP1 binding partner TRAF3 as a positive control [53]. We observed a strong association between LMP1 and BCL10, and weaker interactions with CARMA1 and MALT1, possibly mediated by endogenous BCL10 (Fig. 5A). Mutation of the CTAR1 motif reduced BCL10 binding to background levels, while CTAR2 mutation had no effect (Fig. 5B). Since both CTAR mutations affect MALT1 activation (Fig. 4B), factors other than BCL10 binding must contribute to LMP2-driven MALT1 activation. Physical interaction of LMP1 with BCL10 was confirmed at the endogenous level, by co-immunoprecipitation of the two proteins in VAL and VL51 cell lines (Fig. 5C). BCL10 binding to LMP1 was detectable in both MALT1-active and -inactive cell lines (Fig. 5C), suggesting that LMP1-mediated recruitment of BCL10 was not sufficient for MALT1 activation. Endogenous BCL10 could not be reliably detected by microscopy, but endogenous MALT1 and LMP1 showed partial co-localization by microscopy (Fig. 5D).

Finally, we tested whether LMP2 proteins can interact with CBM components upon co-expression in 293T cells. In lysates of transfected cells, LMP2A and LMP2B proteins were detectable as monomers and SDS-resistant oligomers by SDS-PAGE and western blot. Despite weaker expression of LMP2B compared to LMP2A, co-immunoprecipitation experiments revealed a strong interaction of both LMP2 isoforms with MALT1, including both monomeric and higher molecular weight forms of LMP2A/B (Fig. 5E). These findings suggest that LMP1 and LMP2 recruit BCL10 and MALT1, respectively, and most likely activate MALT1 through formation of oligomeric LMP1-BCL10-MALT1 (LBM) complexes.

### MALT1 inhibition impairs NF-κB-driven gene expression in EBV-transformed cells lines

**Fig. 6:**
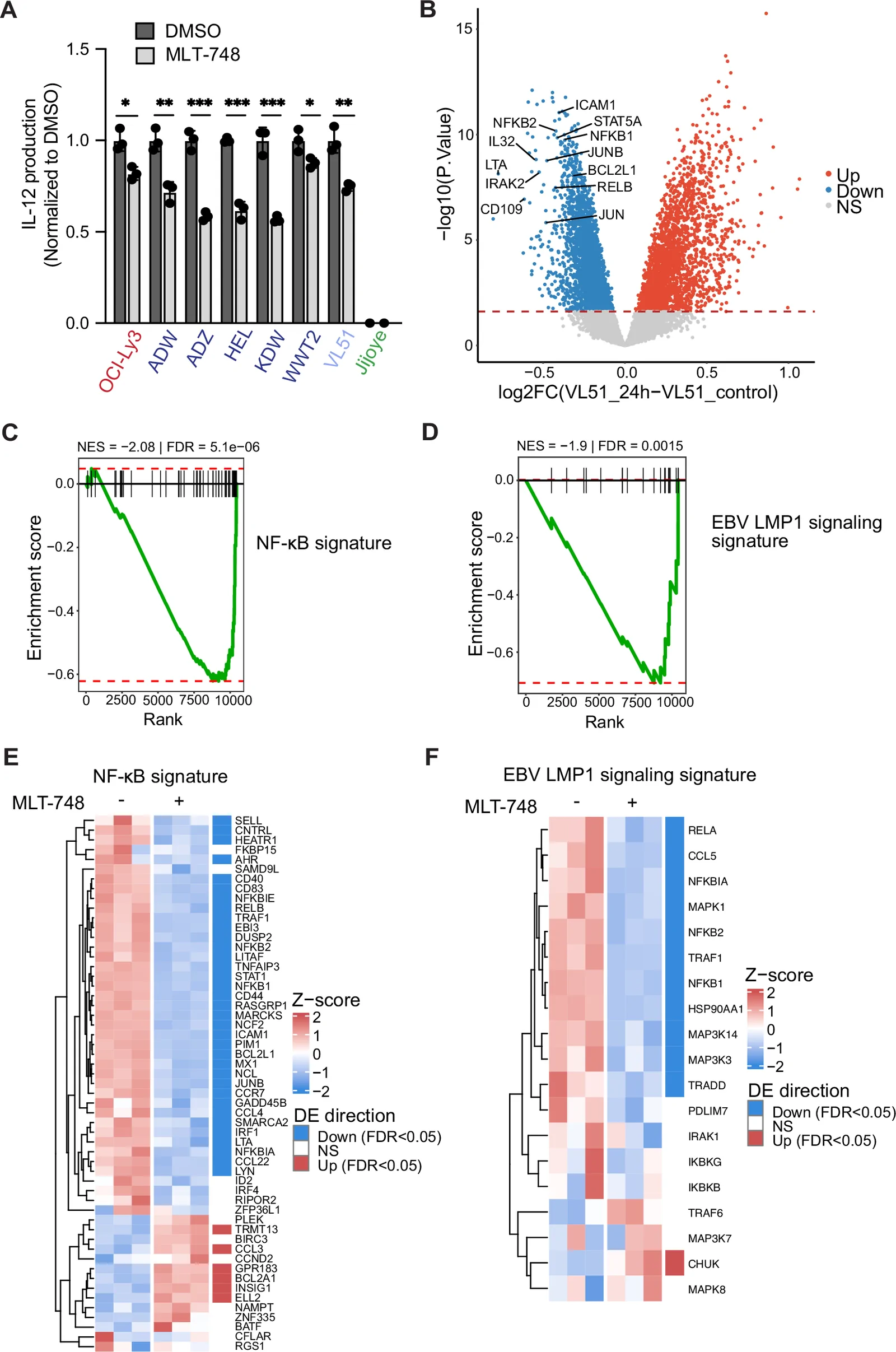
Effect of MALT1 inhibition on gene expression in EBV^+^ lymphoma models. **A** The indicated lymphoma cell lines were treated for 48 h with the MALT1 inhibitor MLT-748 or solvent (DMSO), and levels of IL-12 in the culture supernatant were assessed by ELISA. **B** Volcano plot showing differentially expressed genes that are significantly upregulated (red) or downregulated (blue) upon treatment of VL51 cells for 24 h with the MALT1 inhibitor MLT-748. **C**, **D** Gene set enrichment analysis (GSEA) using previously curated gene lists related to NF-κB signaling (“NFkB_Up_all_OCILy3_Ly10”) [54] and EBV LMP1 signaling (MSigDB accession M39410; WP262). **E**, **F** Heatmaps for individual genes of the gene signatures in (**C**), for 55/65 genes, and (**D**), for 19/23 genes. A right-hand annotation bar indicates the direction of differential expression for the MLT-748-treated versus control contrast (red, up-regulated; blue, down-regulated; white, not significant at FDR < 0.05).

Having established a role for MALT1 in LMP1- and LMP2-driven NF-κB activation, we proceeded to assess the effect of MALT1 inhibition on expression of known NF-κB target genes and, more generally, on the gene expression pattern of EBV-transformed cell lines. As cytokines are crucial for B-cell growth and survival, we first monitored their secretion in EBV-transformed cell lines (Supplementary Fig. 2A–D). IL-12, a known NF-κB target, was secreted in most EBV^+^ cell lines (ADW, ADZ, HEL, KDW, WWT2, VL51) (Supplementary Fig. 2A), and we therefore assessed the effect of MALT1 inhibition on IL-12 secretion in these lines. The MALT1 inhibitor MLT-748 showed a strong reduction in the production of IL-12 (Fig. 6A). To obtain a broader picture of MALT1-driven gene expression in EBV-transformed cell lines, we then treated the splenic marginal zone B-cell lymphoma cell line VL51 with the MALT1 inhibitor MLT-748 for 24 h and analyzed the resulting effects on gene expression by an RNA-Seq approach. This analysis revealed changes in gene expression in a total of 5’204 genes (Supplementary Fig. 3A). GSEA Hallmark analysis performed on the full list of differentially expressed genes identified ‘TNF signaling via NF-κB’ and ‘inflammatory response’ as negatively enriched pathways (Supplementary Fig. 3B). Amongst the most significantly downregulated genes were several previously identified NF-κB target genes (Fig. 6B), including ICAM1, BCL2L1 and c-Jun, which we validated by RT-PCR (data not depicted). Further GSEA analysis and gene expression profiling using previously described NF-κB gene expression signatures [54] revealed a clear negative enrichment score for these signatures, as well as for signatures describing EBV LMP1 signaling (MSigDB accession M39410; systematic name WP262) and LMP1 early responses (MSigDB accession M13941) [55] (Fig. 6C–F, Supplementary Fig. 3C–F). Collectively, these findings suggest that MALT1 protease activity controls expression of LMP1- and NF-κB-driven genes in EBV-transformed cells lines.

### MALT1 inhibition impairs growth of EBV-transformed, LMP1^+^LMP2^+^ B cell lines in vitro and in vivo

**Fig. 7:**
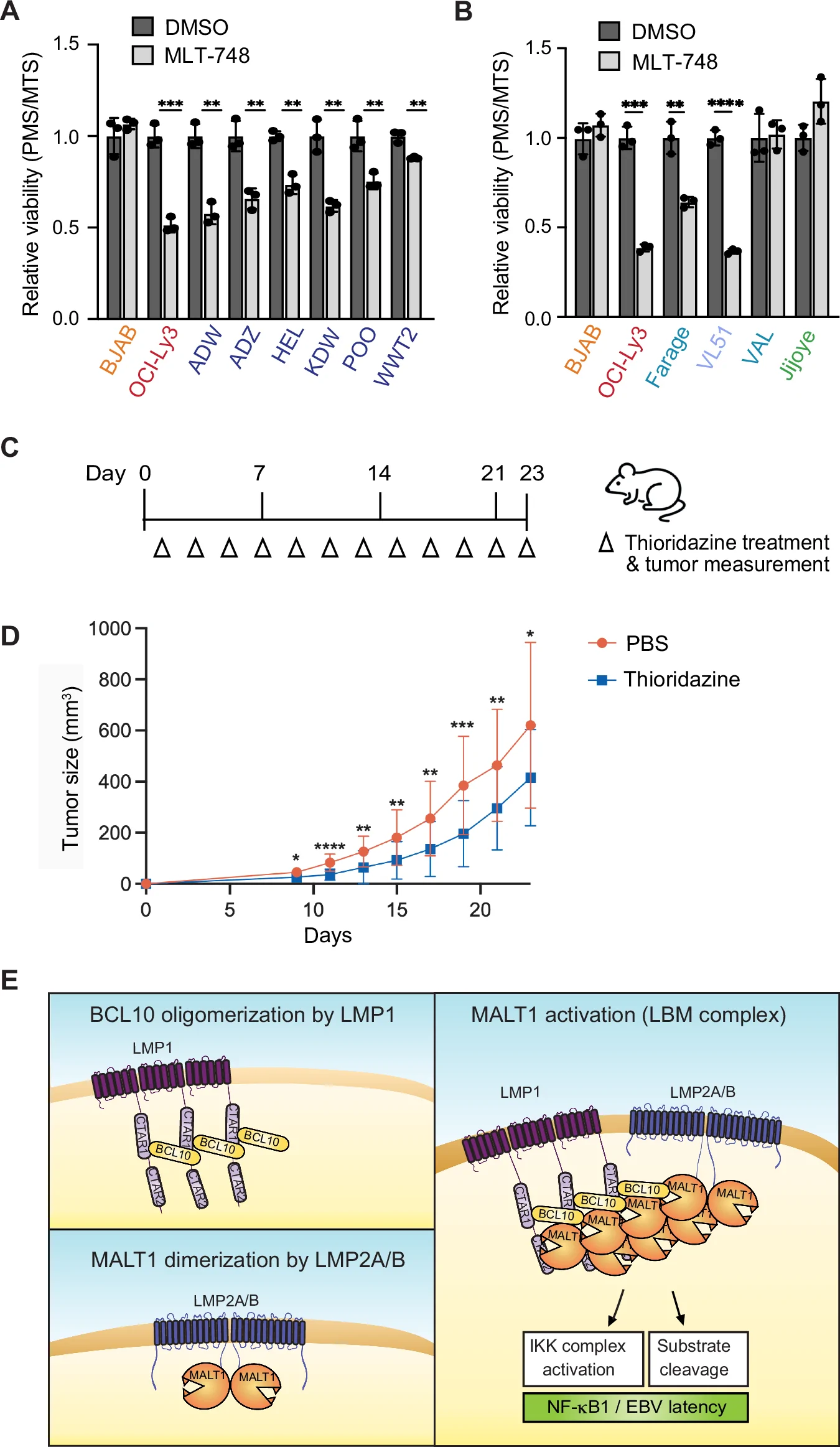
MALT1 inhibition impairs growth of EBV^+^ lymphoma models in vitro and in vivo. **A**, **B** The indicated B-cell lymphoma cell lines were treated with the allosteric MALT1 inhibitor MLT-748 or solvent alone (DMSO). Cell viability was assessed using MTS/PMS assay 7 days after treatment. Data in (**A**, **B**) are representative of two independent experiments. **C** Overview of the time course of the Farage xenograft model. Farage cells were injected into the flanks of mice on day 0. Arrowheads indicate time points at which mice were treated with the MALT1 inhibitor Thioridazine or solvent alone, and when tumor size was monitored using a caliper. **D** Analysis of the overall tumor size of Farage-injected animals under conditions of treatment with vehicle (PBS) or the MALT1 inhibitor Thioridazine. Data pooled results from two independent experiments using 10 mice per condition. **E** Hypothetical model for LMP1- and LMP2-mediated MALT1 activation by LMP1-mediated BCL10 oligomerization, LMP2A/B-mediated MALT1 dimerization and formation of an LMP-BCL10-MALT1 (LBM) complex.

To determine if MALT1 activity is essential for the survival of EBV-infected lymphoma cell lines with LMP1 and LMP2 expression, we treated various EBV-transformed cell lines with the allosteric MALT1 inhibitor, MLT-748. MALT1 inhibitor treatment led to a clear decrease of cell viability in EBV-transformed LCLs, such as ADZ, ADW, HEL, KDW, POO and WWT2 (Fig. 7A) and in the EBV^+^ lymphoma cell lines Farage and VL51 (Fig. 7B), which are characterized by concomitant LMP1 and LMP2 expression (Fig. 3A, B). Similar effects were observed when cells were treated with the irreversible MALT1 inhibitor z-LVSR-fmk [48] or the clinically advanced MALT1 inhibitor compound Safimaltib [56] (Supplementary Fig. 4A–B). The ABC DLBCL cell lines OCI-Ly3, TMD8 or HBL1, used as a positive control, also showed impaired growth with MALT1 inhibition (Fig. 7B, Supplementary Fig. 4). In contrast, MALT1 inhibition did not affect the viability of EBV^+^ lymphoma cell lines that were LMP2-negative and lacked constitutive MALT1 activity (Fig. 7B, Supplementary Fig. 4A–B), such as the DLBCL cell line VAL and the Burkitt’s lymphoma cell line Jijoye, or the EBV-negative DLBCL line BJAB (see Fig. 3A, B). Thus, MALT1 protease activity is crucial for the growth of LMP1^+^LMP2^+^ lymphoma cell lines, but not for LMP1^+^LMP2^-^ cell lines including Burkitt lymphoma cell lines and the DLBCL cell line VAL.

Finally, to evaluate MALT1 inhibition as a treatment option for EBV^+^ B cell lymphomas with LMP1 and LMP2 co-expression, we tested the effect of the MALT1 inhibitor Thioridazine on the growth of the EBV^+^ DLBCL cell line Farage in a xenograft model. Farage cells were injected into the flanks of NOD/SCID mice, and after 24 h, mice were treated with Thioridazine (10 mg/kg) or vehicle every 48 h for 23 days (Fig. 7C). Tumor outgrowth was significantly delayed in Thioridazine-treated mice compared to controls (Fig. 7D). This suggests that MALT1 inhibition by Thioridazine effectively delays the growth of EBV^+^ lymphoma cells in vivo.

## Discussion

Our findings provide several lines of evidence supporting a critical role for LMP-mediated MALT1 activation in promoting the growth of EBV^+^ B-cell lymphomas. First, the biochemical and microscopic data support a model in which oligomeric LMP1 and LMP2 proteins recruit BCL10 and MALT1, respectively, forming oligomeric LMP-BCL10-MALT1 (LBM) complexes that activate MALT1 in a CBM-like manner. Moreover, we showed that LBM-mediated MALT1 activation occurred independently of the kinases BTK and PKCβ, which are required for BCR-induced MALT1 activation through CARMA1 phosphorylation. Finally, we observed that MALT1 inhibition efficiently reduced tumor growth in vitro and in vivo, suggesting use of MALT1 inhibitors as a treatment option for EBV^+^ B-cell lymphomas with LMP1-LMP2 co-expression.

How do LMP1 and LMP2A/B promote MALT1 activation? Our findings support a model in which LMP1 and LMP2 colocalize, as previously reported [57], and physically recruit BCL10 and MALT1, respectively, to favor MALT1 activation in a synergistic manner (Fig. 7E). LMP1, which is known to form oligomeric complexes [7, 58], likely nucleates oligomerization of the scaffold protein BCL10, in a manner similar to the BCL10 fiber-nucleating protein CARMA1 [33]. LMP2A and LMP2B, on the other hand, are known to form homo- and heterodimers [20, 59] that may facilitate dimerization of MALT1. By colocalizing with LMP1, LMP2 may deliver preformed MALT1 dimers onto BCL10 oligomers and/or favor dimerization of MALT1 concurrently with its incorporation into BCL10 oligomers, facilitating proximity-induced MALT1 activation. Thereby, LMP1 and LMP2 may nucleate LBM complexes that resemble CBM complexes involved in MALT1 activation [31, 33]. This model will need to be better defined in the future through additional characterization of the underlying binding motifs in the LMP, MALT1 and BCL10 proteins, and the use of cryo-electron microscopy imaging [7].

We further examined the individual contributions of LMP1 and LMP2 to MALT1 activation. LMP1 expression was sufficient to induce MALT1 protease activity, while LMP2A and LMP2B had minimal effects on their own. However, co-expression of LMP1 with either LMP2A or LMP2B led to strong synergistic activation of MALT1. Surprisingly, LMP2B, which lacks the N-terminal signaling tail, synergized more effectively with LMP1 than LMP2A, which could reflect either LMP2A’s established role in limiting B-cell activation or an increased efficiency of LMP2B in binding to MALT1. Our mutational analysis showed that LMP1 recruits BCL10 through its CTAR1 motif, which is known to bind to TRAF family members [9]. In our hands, co-expression of TRAF proteins did not enhance the LMP1-BCL10 interaction (data not depicted), suggesting that BCL10’s recruitment to the LMP1 CTAR1 motif occurs independently of TRAF family members. Recent analysis of LMP1-mediated oligomerization by cryo-electron microscopy suggests that LMP1 assembles into filamentous oligomers that can recruit TRAF3 trimers for signaling [7]. How BCL10 recruitment by the cytoplasmic CTAR1 motif of LMP1 might affect this higher order assembly will be an interesting topic of future studies.

A key finding of our work is that MALT1 plays a major role in LMP1- and LMP2A/B-driven NF-κB activation, which is critical in various lymphomas. Targeting critical drivers of this pathway, such as MALT1, therefore offers a promising therapeutic strategy. While ABC DLBCL rely on BCR-BTK-PKC-CARMA1 signaling for MALT1 and NF-κB activation and can thus be targeted with the BTK inhibitor Ibrutinib [60], we find that EBV^+^ cell lines activate MALT1 directly, via LMP1 and LMP2, independently of BTK and PKC. This is reminiscent of our previously published observation that the K13 and K15 proteins of another lymphotropic herpesvirus, KSHV, activate MALT1 in a BTK-, PKC- and CARMA1-independent manner [40]. We conclude that both, EBV and KSHV promote MALT1 activation using unique viral proteins that either physically interact with MALT1 (such as K13 and LMP2) or activate MALT1 by indirect means (e.g., K15 and LMP1) that remain to be fully characterized. Thus, use of MALT1 inhibitors could be a rational treatment option not only for KSHV-driven malignancies, but also for EBV^+^ lymphomas with concurrent LMP1 and LMP2 expression and constitutive MALT1 activity. Our data based on cell lines and a xenograft model with the EBV^+^ Farage cell line suggest that MALT1 inhibition can indeed slow down the growth of such tumor cells in vitro and in vivo. It is possible that advanced MALT1 inhibitor compounds, which have entered clinical trials [61, 62], would show more pronounced effects in vivo, and this should be tested in future studies. MALT1 inhibitors may additionally stimulate viral lytic reactivation, offering a potential strategy for eliminating EBV infection. Clinically validated MALT1 inhibitors should therefore be considered for treatment of EBV^+^ lymphoma, possibly in combination with strategies targeting EBV replication, such as nucleoside analogs [2]. Collectively, our findings identify MALT1 as a driver of EBV-mediated cellular transformation in lymphomas characterized by EBV latency stage II or III and therefore identify MALT1 as a potentially interesting target for the treatment of EBV-driven malignancies.

## Supplementary information

Supplemental data

## Data Availability

The datasets generated and analysed during the current study (RNA-sequencing data for the six VL51 libraries) have been deposited in the GEO repository under accession number GSE335838.
