# Supplementary material for: A role for the protease MALT1 in EBV-mediated B-cell transformation by Latent Membrane Proteins 1 and 2
Source: Leukemia. 2026 Aug 13;40(11):2455–65. doi: 10.1038/s41375-026-03109-8 (PMC13713546; doi:10.1038/s41375-026-03109-8)
Supplement: Supplementary file 1 [file 41375_2026_3109_MOESM1_ESM.pdf]

Supplementary Information

A role for the protease MALT1 in EBV-mediated B-cell transformation by Latent Membrane Proteins 1 and 2

Mélanie Juilland, Laurence Romy*, Harun-Or Rashid*, Sara Escudeiro Lopes*, Silvia Podavini*, David Vallois, Manhui Luo, Montserrat Gonzalez, Katerina Pandeva, Frédéric Frête, Chantal Décaillet, Laurence de Leval, Sylvia Rothenberger and Margot Thome

Summary:

The supplementary information file contains supplementary material and methods, four supplementary figures, a supplementary table and four supplementary references.

Supplementary Material and Methods

Protein expression vectors

Expression vectors for wild-type and CTAR1- or CTAR2-mutated LMP1 have been previously described [1]. The CTAR1/2 double mutant was generated by introducing the CTAR2 point mutation (L382A), identical to that used in the CTAR2 single mutant, into the CTAR1 single mutant background, using the primers F= 5'-CCA CGG CCC AGT TCA GGC AAG CTA CTA TGA CTA A-3' and R= 5'-TTA GTC ATA GTA GCT TGC CTG AAC TGG GCC GTG G-3'. LMP2A and LMP2B sequences were cloned into PCR3.1 and the eYFP–Leu-Val-Ser-Arg–eCFP MALT1 reporter construct has been previously described [2].

Cell viability assay

Cells (2.5×10^5/ml) were treated with 10 µM of MLT-748 (MedChemExpress, Sollentuna, Sweden), 10 µM of z-LVSR-fmk (Bachem, Bubendorf, Switzerland), 10 or 20 µM of Safimaltib (MedChemExpress/Lucerna-Chem AG, Luzern, Switzerland) or vehicle alone (PBS or DMSO) for 7 days, and cell viability was assessed using at 400 µg/ml of 3-(4,5-dimethylthiazol-2-yl)-5-(3-carboxymethoxyphenyl)-2-(4-sulfophenyl)-2H-tetrazolium (MTS, Promega, Dübendorf, Switzerland) and 9 µg/ml of phenazine methosulfate (PMS, Merck, Buchs, Switzerland), according to the manufacturer's instructions. Reduction of MTS to formazan was measured at 492 nm with Capture 96 Software on a Ledetect 96 microplate spectrophotometer (Dynamica Scientific, Livingston, UK).

Cell Preparation and Immunofluorescence Staining

Cells were cultured in 12- or 24-well plates and subsequently transferred onto coverslips pre-treated with poly-L-lysine (CAS No: 25988-63-0, Merck, Buchs, Switzerland) for 1h. The

plates were centrifuged at 1200 RPM for 3 min, after which they were returned to the incubator for an additional 1h to allow for adhesion. Following this, the cells were fixed using 4% paraformaldehyde (PFA) solution for 15 min on ice. Permeabilization was performed using permeabilization buffer (0.5% [w/v] saponin + 0.5% [w/v] BSA in PBS) for 30 min. Cells were then incubated with primary mouse anti-MALT1 and anti-LMP1 antibodies diluted in the permeabilization buffer for 1 hour, followed by incubation with Alexa Fluor-conjugated isotype-specific secondary antibodies, also diluted in the permeabilization buffer, for 45 min. The MALT1 antibody is of isotype IgG1α, detected by goat anti-mouse IgG1α Alexa Fluor 594 conjugate (Cat# A21125, Invitrogen/Thermofischer, Reinach, Switzerland), while the LMP1 antibody is an IgG2α, detected by goat anti-mouse IgG2α Alexa Fluor 488 conjugate (Cat# A21131, Invitrogen/Thermofischer, Reinach, Switzerland). Microscopy images were acquired using an inverted Zeiss LSM 800/880 confocal microscope equipped with a Plan Apochromat 63×/1.40 NA oil immersion DIC II objective lens. For colocalization analysis, the JACoP plugin in ImageJ software was used.

NF-κB luciferase reporter assay

Activation of gene transcription by LMP1, LMP2A, or LMP2B was assessed by transient transfection of control or MALT1-silenced HEK293T cells with a NF-κB firefly luciferase reporter construct together with a Renilla luciferase vector (phRL-TK). Cells were lysed in passive lysis buffer (Promega, Dübendorf, Switzerland) and luciferase activity was assessed using dual luciferase assay (Promega, Dübendorf, Switzerland) on a Spectramax ID5 instrument using an injector in a 384 q-PCR plate format.

Quantitative real-time PCR

Total RNA from BJAB, Raji, Jijoye, VAL, Farage, VL51, HEL, KDW, NDW and WWT2 cell lines was extracted with TRIzol reagent (Life Technologies/Thermofischer, Reinach, Switzerland) according to manufacturer's instructions and cDNA was synthesized with a High-Capacity cDNA Reverse Transcription kit (Applied Biosystems/Thermofischer, Reinach, Switzerland). SYBR Green fluorescent reagent and LightCycler480 Real Time PCR System (Roche, Rotkreuz, Switzerland) were used for quantitative RT-PCR with the following cycling conditions: 10 seconds pre-incubation at 95 °C, followed by 50 amplification cycles consisting each of denaturation at 95 °C, annealing at 55 °C and extension at 72 °C followed by generation of the melting curve. The relative amount of mRNA was calculated by the comparative threshold cycle method with the housekeeping gene GAPDH (forward: 5'-GAAGGTGAAGGTCGGAGT-3', reverse: 5'-GAAGATGGTGATGGGATTTC-3'). Primers for LMP2A (forward: 5'-AGGTACTCTTGGTGCAGCCC-3' and reverse: 5'-AGCATATAGGAACAGTCGTGCC-3'), LMP2B (forward: 5'-CGGGAGGCCGTGCTTTAG-3' and reverse: 5'-GGCGGTCACAACGGTACTAACT-3'), EBNA2 (forward: 5'-ACGCATTAGAGACCACTTTGAGCC-3' and reverse: 5'-AAGCGCGGGTGCTTAGAAGG-3') EBNA LP (forward: 5'-CCCCTCTCTCTGTCCTTCAG-3' and reverse: 5'-GGCTCCCCTCAGACATTCTT-3'), BLZF1 (forward: 5'-TTCCACAGCCTGCACCAGT-3' and reverse: 5'-AGCAGCCACCTCACGGTAGT-3') and BHFR1 (forward: 5'-GTCAAGGTTTCGTCTGTGTG-3' and reverse: 5'-TTCTCTTGCTGCTAGCTCCA-3')

Deep sequencing and mutation analysis

Three cell lines were examined by next-generation sequencing (NGS). Data were generated by Targeted Deep Sequencing. 500 ng of DNA, extracted using the Maxwell instrument (Promega,

Dübendorf, Switzerland), were fragmented with the KAPA HyperPlus kit (Roche, Pleasanton, CA, US) and target-enriched by capture using the xGen® Lockdown® IDT (Integrated NA Techologies, Newark, NJ, USA) for a custom B-cell lymphoma gene panel developed in house. This panel targeted the full coding sequences of 54 genes of interest involved in mature B-cell lymphoma (ARID1A, BCL10, CD58, ID3, NOTCH2, PIK3CD, TNFRSF14, CXCR4, REL, SF3B1, XPO1, BCL6, MYD88, CCND3, IRF4, NFKBIE, PRDM1, TNFAIP3, BRAF, CARD11, EZH2, KMT2C, MYC, CDKN2A, CD274, NOTCH1, PDCD1LG2, TRAF2, PTPRD, PTEN, BIRC3, ATM, CCND1, CDK4, KMT2D, STAT6, FOXO1, B2M, MAP2K1, CREBBP, PLCG2, SOCS1, CIITA, CD79B, GNA13, MAP3K14, TP53, BCL2, CD79A, KLF2, MEF2B, TCF3, EP300, BTK). Sequencing was performed on a MiSeqTM System (Illumina). Alignment was performed using BWA software with default setting and the Human genome GRCh37 assembly. Variant calling, including Single Nucleotide Variants (SNVs), small insertions and deletions (InDels), was performed using VarScan2 with the following parameters: minimum coverage > 100; minimum ALT reads > 50; minimum variant frequency >1%; minimum average base quality 20; bias in strand for reads supporting variant <0.05. A mix of DNA from B-cell lymphoma cell lines with known mutations was used as control.

Analysis of MALT1 protease activity by flow cytometry

Forster resonance energy transfer (FRET)-based determination of MALT1 protease activity in HEK293T cells was performed as described (Pelzer *et al.*, 2013). In brief, HEK293T cells were transfected with the eYFP–Leu-Val-Ser-Arg–eCFP reporter construct, together with indicated combinations of LMP1, LMP1 CTAR1/2 single or double mutants, LMP2A, LMP2B, MALT1 and BCL10 expression constructs. Dominant-negative mutant of I kappa B alpha (IκBα-S32A/S36A) was co-expressed in all the conditions to prevent CMV promotor activation by NF-κB. After 6h of transfection, cells were washed and 24h later resuspended in flow

cytometry buffer (2% fetal bovine serum in phosphate-buffered saline) and analyzed with an LSR II SORP flow cytometer (BD Biosciences, Allschwill, Switzerland) containing 405, 488, 561 and 640 nm lasers. To measure the eCFP and Forster resonance energy transfer signal, the transfected cells were excited with a standard 450/50 filter for collection of the eCFP fluorescence and a 585/42 filter for Forster resonance energy transfer fluorescence, respectively, and for each sample at least 10’000 highly eYFP+ cells were counted (Pelzer *et al.*, 2013).

Analysis of gene expression by RNA sequencing

VL51 cells were treated for 24h with 10 µM of MLT-748 or solvent alone (DMSO), and total RNA was extracted with RNeasy Plus Mini Kit according to the manufacturer’s protocol (Qiagen, ID 74134). The quality of the extracted RNA was assessed on a Fragment Analyzer (Agilent Technologies). RNA-seq libraries were prepared from 250 ng of total RNA with the Watchmaker mRNA Library Prep Kit (Watchmaker Genomics) using a unique dual indexing strategy and following the official protocol. Libraries were quantified by a fluorometric method (QubIT, Life Technologies) and their quality assessed on a Fragment Analyzer (Agilent Technologies). Sequencing was performed on an Element Biosciences Aviti with a 2x75 cycles sequencing kit Cloudbreak FS High Output (run as single read 150). Base calling and demultiplexing was done with bases2fastq version: 2.0 with filter mask R1:N15Y15N. Statistical analysis was performed at the gene level in R (v4.3.3) using edgeR. Differential expression was assessed using the limma-trend approach on log2-CPM values. Moderated t-statistics were computed with empirical Bayes shrinkage, and p-values were adjusted by the Benjamini–Hochberg method. Ggenes with an FDR < 0.05 and |log2FC| > 1 were considered differentially expressed (5'204 genes), and visualized in a volcano plot of log2 fold change versus −log10(adjusted p-value).

Gene set enrichment analysis was performed with fgsea (v1.28.0) on the full ranked list of expressed genes. Genes were ranked by a signed metric, rank = sign(log2FC) × −log10(p-value), placing strongly and significantly regulated genes at the extremes of the ranking. fgsea was run with a minimum gene set size of 5, 10'000 permutations, and a fixed seed (set.seed(42)), with p-values adjusted by the Benjamini–Hochberg method. Two gene set sources were tested. First, the Hallmark collection (50 gene sets) was retrieved from MSigDB via the msigdbr package (v26.1.0); significant pathways (FDR < 0.05) with negative NES were displayed as a bubble plot (ggplot2 v3.5.1), with the normalised enrichment score (NES) encoded by colour and −log10(FDR) by point size. Second, curated gene sets related to NF-κB signaling and EBV LMP1 response, assembled from published studies and pathway databases, were tested, and enrichment plots for the four presented pathways were generated with the plotEnrichment function. The NF-κB signatures used were the Staudt Lab Signatures (StaudtSigDB): "NFkB_Up_all_OCILy3_Ly10" and "NFkB_Up_bothOCILyandLy10" [3]. For analysis of genes induced by EBV LMP1 signaling, we used the "WP_EBSTEINBARR_VIRUS_LMP1_SIGNALING" gene set (MSigDB accession M39410; WP262), sourced from WikiPathways and accessed via MSigDB C2 canonical pathways collection, version 2026.1.Hs (May 2026)), and the "DIRMEIER_LMP1_RESPONSE_EARLY" gene set (MSigDB accession M13941) [4], accessed via the MSigDB C2 curated gene sets collection. For expression visualization, the genes from selected pathways present in the filtered dataset were matched by gene symbol and z-scored per gene across samples, then displayed as heatmaps with ComplexHeatmap (v2.18.0). The six VL51 libraries were ordered by time point, and genes were clustered hierarchically by Euclidean distance. The 5'204 differentially expressed genes were displayed as a separate heatmap of per-gene z-scored log2-CPM values using the same settings. Library preparation and sequencing was performed at the Lausanne Genomic Technologies Facility,

University of Lausanne, Switzerland (https://www.unil.ch/gtf/en/home.html). RNA-sequencing data for the six VL51 libraries have been deposited in GEO under accession number GSE335838.

Xenograft Experiments

NSG mice were maintained under pathogen-free conditions in the animal facility of the University of Lausanne. All mouse experiments were authorized by the Swiss Federal Veterinary Office. For xenograft studies, NSG mice were inoculated with 10^7 Farage cells diluted in 200 µL sterile PBS into the flanks of the animals. The MALT1 inhibitor Thioridazine (10 mg/kg body weight), or vehicle alone, was administered using an insulin syringe for i.p. injection. Drug was administered 24h after Farage injection, 3 times/week. Two experiments, with 10 mice per group for each experiment, were performed. Tumor volume was determined by digital caliper measurements and calculated by using the formula (length × width squared)/2. Investigators were not blinded to the group allocation and no randomization was used.

Supplementary Figures

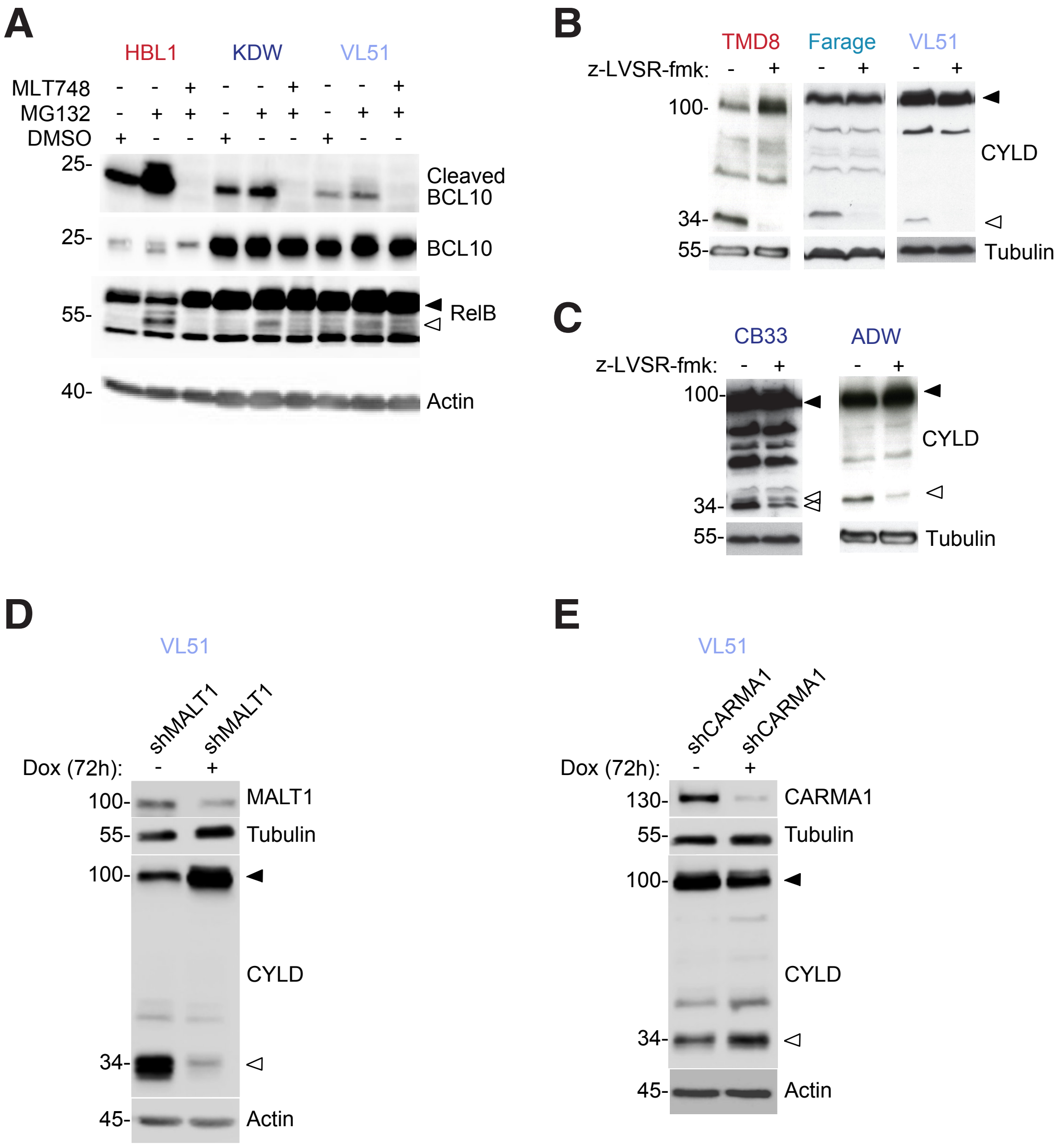

Figure S1. Effect of MALT1 inhibition and MALT1 or CARMA1 silencing on substrate cleavage in EBV-transformed B-cell lines

(A) Immunoblot using antibodies directed against BCL10, cleaved BCL10 and RelB was used to monitor substrate cleavage in lysates of an ABC DLBCL (HBL-1), an EBV-positive LCL (KDW) and an EBV-positive SMZL (VL51). Treatment with the MALT1 inhibitor MLT-748 for 24h was used to inhibit substrate cleavage, and MG132 was added for the last 3h of treatment to allow for accumulation of cleaved RelB and BCL10. β-Actin served as a control

for equal loading. (B, C) Immunoblot for CYLD was used to monitor MALT1 activity in lysates of ABC DLBCL (TMD8), EBV-positive GCB DLBCL (Farage), EBV-positive LCL (ADW, CB33), EBV-positive SMZL (VL51), treated with solvent (DMSO) or z-LVSR-fmk. (D, E) Cleavage of CYLD was monitored upon doxycycline-induced silencing of MALT1 or CARMA1 expression in the stated cell lines. Tubulin and β-Actin served as controls for equal loading. Cleaved and uncleaved protein species are indicated with open and black arrowheads, respectively. (A-E) Data are representative of two independent experiments.

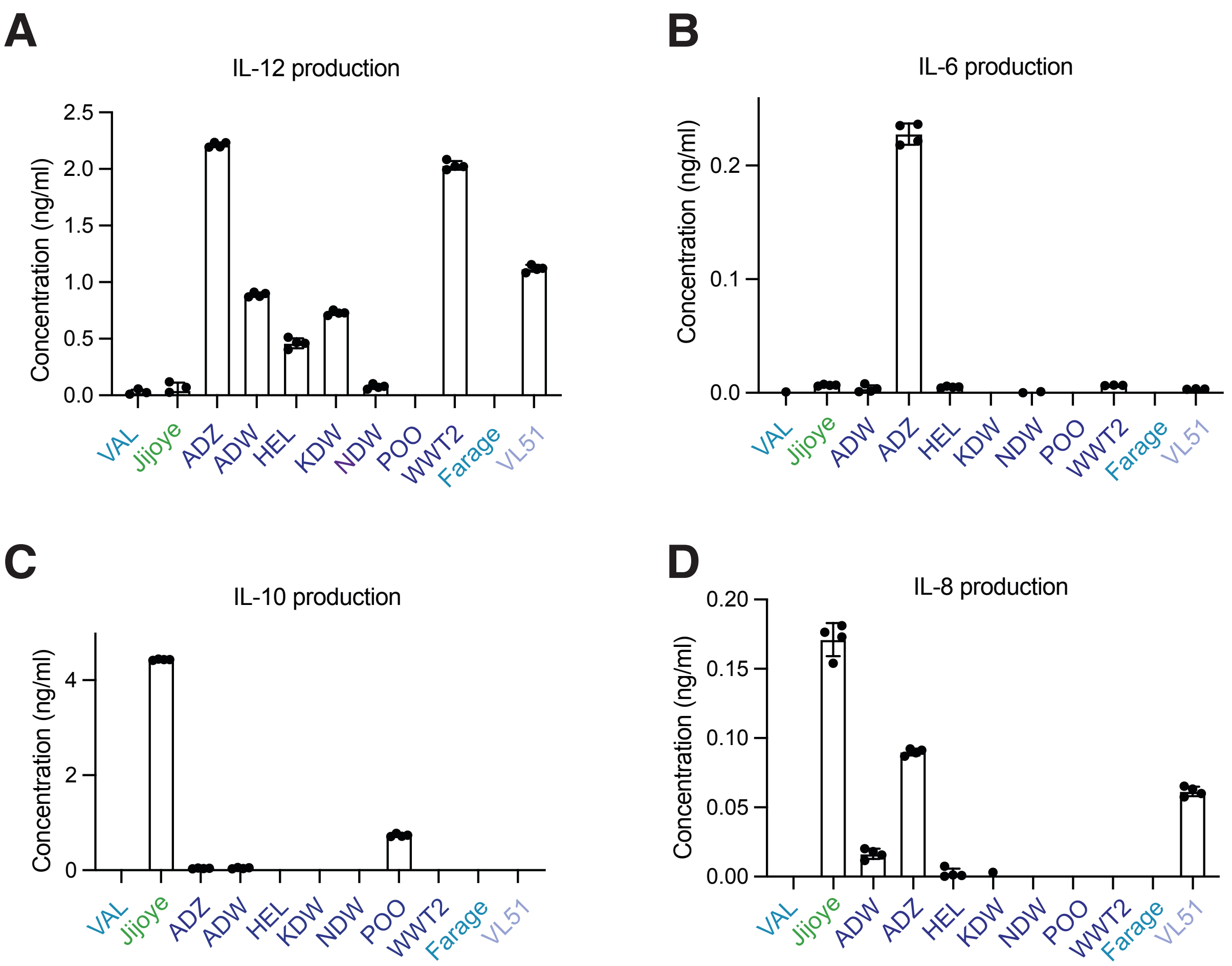

Figure S2. Cytokine production in EBV positive B-cell lines.

(A-D) The indicated EBV positive B-cell lines were seeded for 48h, and levels of IL-12 (A), IL-6 (B), IL-10 (C) or IL10 (D) in the culture supernatant were assessed by ELISA. (A-D) Data are representative of two independent experiments.

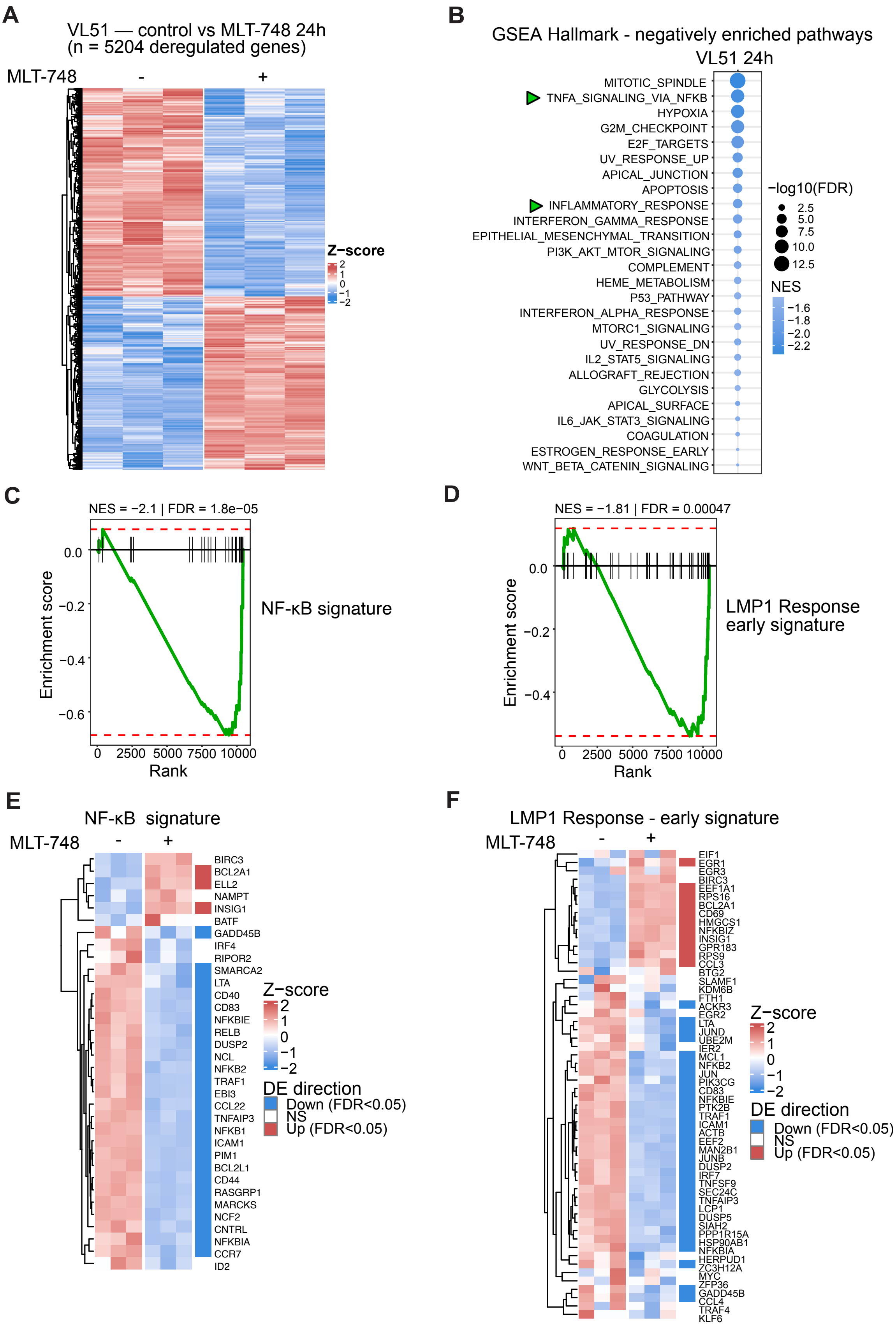

A
VL51 — control vs MLT-748 24h
(n = 5204 deregulated genes)
MLT-748
-
+
Z-score
2
1
0
−1
−2
B
GSEA Hallmark - negatively enriched pathways
VL51 24h
MITOTIC_SPINDLE
TNFA_SIGNALING_VIA_NFKB
HYPOXIA
G2M_CHECKPOINT
E2F_TARGETS
UV_RESPONSE_UP
APICAL_JUNCTION
APOPTOSIS
INFLAMMATORY_RESPONSE
INTERFERON_GAMMA_RESPONSE
EPITHELIAL_MESENCHYMAL_TRANSITION
PI3K_AKT_MTOR_SIGNALING
COMPLEMENT
HEME_METABOLISM
P53_PATHWAY
INTERFERON_ALPHA_RESPONSE
MTORC1_SIGNALING
UV_RESPONSE_DN
IL2_STAT5_SIGNALING
ALLOGRAFT_REJECTION
GLYCOLYSIS
APICAL_SURFACE
IL6_JAK_STAT3_SIGNALING
COAGULATION
ESTROGEN_RESPONSE_EARLY
WNT_BETA_CATENIN_SIGNALING
−log10(FDR)
2.5
5.0
7.5
10.0
12.5
NES
−1.6
−1.8
−2.0
−2.2
C
NES = −2.1 | FDR = 1.8e−05
Enrichment score
0.0
−0.2
−0.4
−0.6
0
2500
5000
7500
10000
Rank
NF-κB signature
D
NES = −1.81 | FDR = 0.00047
Enrichment score
0.0
−0.2
−0.4
0
2500
5000
7500
10000
Rank
LMP1 Response
early signature
E
NF-κB signature
MLT-748
-
+
BIRC3
BCL2A1
ELL2
NAMPT
INSIG1
BATF
GADD45B
IRF4
RIPOR2
SMARCA2
LTA
CD40
CD83
NFKBIE
RELB
DUSP2
NCL
NFKB2
TRAF1
EBI3
CCL22
TNFAIP3
NFKB1
ICAM1
PIM1
BCL2L1
CD44
RASGRP1
MARCKS
NCF2
CNTRL
NFKBIA
CCR7
ID2
Z−score
2
1
0
−1
−2
DE direction
Down (FDR<0.05)
NS
Up (FDR<0.05)
F
LMP1 Response - early signature
MLT-748
-
+
EIF1
EGR1
EGR3
BIRC3
EEF1A1
RPS16
BCL2A1
CD69
HMGCS1
NFKBIZ
INSIG1
GPR183
RPS9
CCL3
BTG2
SLAMF1
KDM6B
FTH1
ACKR3
EGR2
LTA
JUND
UBE2M
IER2
MCL1
NFKB2
JUN
PIK3CG
CD83
NFKBIE
PTK2B
TRAF1
ICAM1
ACTB
EEF2
MAN2B1
JUNB
DUSP2
IRF7
TNFSF9
SEC24C
TNFAIP3
LCP1
DUSP5
SIAH2
PPP1R15A
HSP90AB1
NFKBIA
HERPUD1
ZC3H12A
MYC
ZFP36
GADD45B
CCL4
TRAF4
KLF6
Z−score
2
1
0
−1
−2
DE direction
Down (FDR<0.05)
NS
Up (FDR<0.05)

Figure S3. Effect of MALT1 inhibition on gene expression in VL51 cells

(A) Heat map showing differentially expressed genes (DEGs, 5'204 total) identified by RNA sequencing of VL51 cells treated for 24h with the MALT1 inhibitor MLT-748 or solvent alone (DMSO). (B) GSEA Hallmark analysis for negatively enriched pathways. Bubble plot displaying the normalized enrichment score (NES) in shades of blue and $-\log_{10}$(FDR) as dot size for the top pathways significantly downregulated. Green arrows highlight pathways for 'TNF signaling via NF-κB' and 'inflammatory response'. (C, D) Gene set enrichment analysis (GSEA) using previously curated gene lists related to NF-κB signaling ("NFkB_Up_bothOCLyandLy10") (ref. 3) and EBV LMP1 early responses (MSigDB accession M13941) (ref. 4). (E, F) Heatmaps for individual genes of the gene signatures in (C), for 34/37 genes, and (D), for 56/62 genes. A right-hand annotation bar indicates the direction of differential expression for the MLT-748-treated versus control contrast (red, up-regulated; blue, down-regulated; white, not significant at FDR < 0.05).

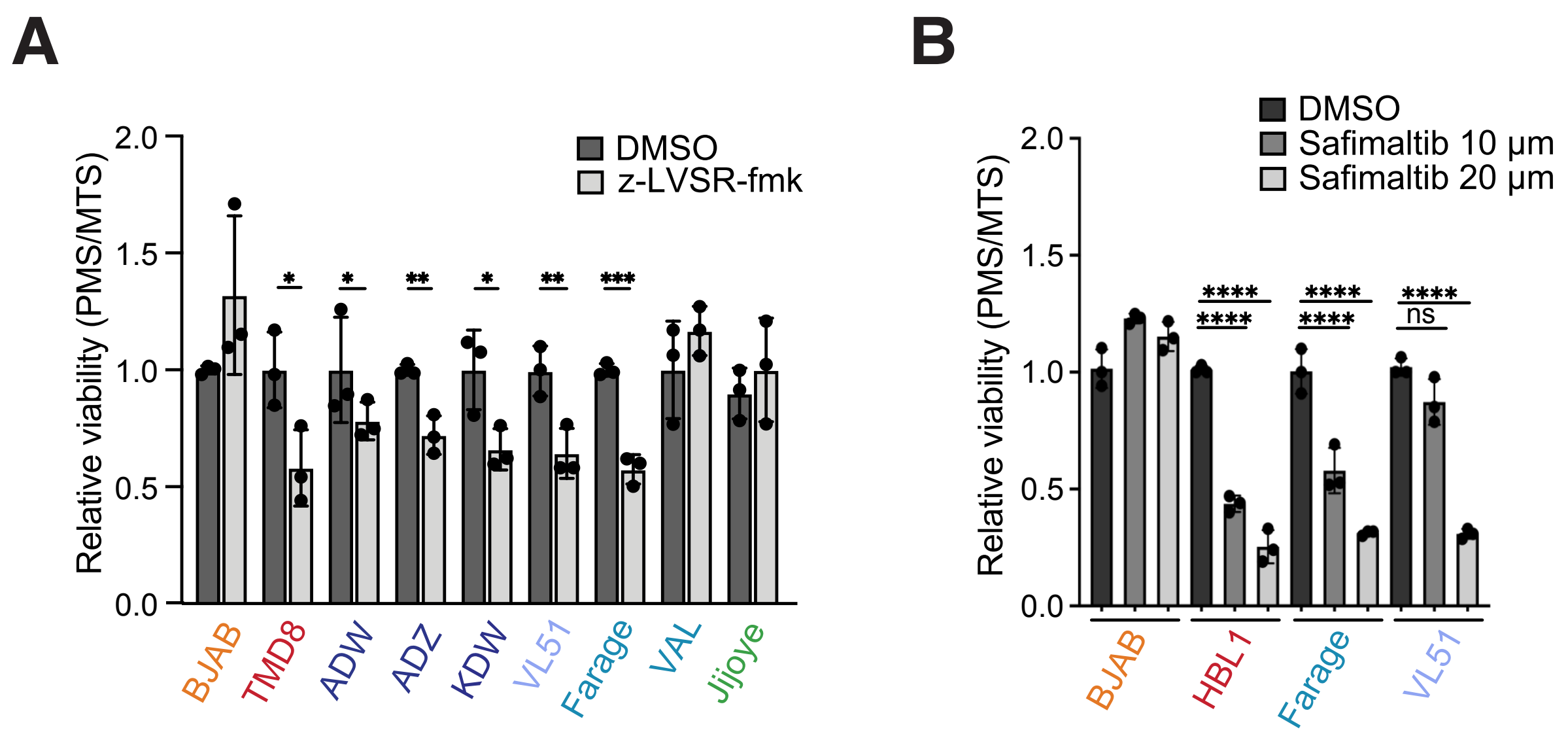

Figure S4. Effect of MALT1 inhibition on growth and viability of EBV-transformed B-cell lines

(A, B) The indicated EBV-transformed B-cell lymphoma cell lines were treated with the tetrapeptide MALT1 inhibitor z-LVSR-fmk (10 µM) (A) or the clinically advanced compound Safimaltib (10 or 20 µM, as indicated) (B). Cell viability was assessed using MTS/PMS assay 7 days after treatment. The GCB-DLBCL model BJAB and the ABC-DLBCL models TMD8 or HBL1 were used as negative and positive controls, respectively, for the effect of the used MALT1 inhibitors.

Supplementary Table

CB33	Gene	Chrom locus	Exon (codons)	Reference	Results	Allele frequency	Coverage	Gen pop (AF)
Mutation	NOTCH2	1p11	1 (0-24)	NM_024408.3	c.-214C>G	18%	2061X	No
Polymorphism	NFKBIE	6p21	1 (0-261)	NM_004556.2	c.285C>G (p.His95Gln)	46%	1423X	Yes (4%)
Polymorphism	KMT2C	7q36	18 (957-992)	NM_170606.2	c.2968A>G (p.Ser990Gly)	7%	1780X	Yes (13%)
Polymorphism	PTPRD	9p24	25 (823-1018)	NM_002839.3	c.2983C>T (p.Arg995Cys)	100%	1196X	Yes (9%)
Polymorphism	NOTCH1	9q34	25 (1338-1529)	NM_017617.4 (LRG_1122)	c.4129C>T (p.Pro1377Ser)	42%	1214X	Yes (2%)
Polymorphism	NOTCH1	9q34	17 (863-913)	NM_017617.4 (LRG_1122)	c.2588-4G>A	42%	1322X	Yes (39%)
Polymorphism	NOTCH1	9q34	11 (557-634)	NM_017617.4 (LRG_1122)	c.1670-9A>G	48%	614X	Yes (36%)
Polymorphism	ATM	11q22	37 (1832-1891)	NM_000051.3 (LRG_135)	c.5557G>A (p.Asp1853Asn)	41%	670X	Yes (14%)
Polymorphism	PLCG2	16q23	9 (231-255)	NM_002661.4 (LRG_376)	(p.His244Arg)	39%	1303X	Yes (4%)
Polymorphism	TCF3	19p13	6 (100-122)	NM_003200.4	c.359T>C (p.Leu120Pro)	47%	1633X	Yes (2%)
Polymorphism	EP300	22q13	15 (939-999)	NM_001429.3	c.2989A>G (p.Ile997Val)	45%	1566X	Yes (29%)
Polymorphism	BTK	Xq22	18 (584-636)	NM_000061.2 (LRG_128)	c.1899C>T (p.Cys633Cys)	100%	201X	Yes (16%)

Supplementary Table S1: Results of CB33, Farage and VL51 genomic DNA analyses.

The table summarizes the results of exon sequencing of the indicated genes. The chromosomal locus (Chrom locus) and the exon analyzed are indicated. The Results column lists the nucleotide mutated and the resulting change in amino acid sequence. Coverage indicates how many times the exon has been read during the analysis. The allele frequencies in the sample and in the general population (Gen pop (AF)) are indicated. LRG, Locus Reference Genomic.

Supplementary References

1. Zuercher, E. *et al.* Genetic diversity of EBV-encoded LMP1 in the Swiss HIV Cohort Study and implication for NF-Kappab activation. *PLoS One* **7**, e32168 (2012).
2. Pelzer, C. *et al.* The protease activity of the paracaspase MALT1 is controlled by monoubiquitination. *Nat Immunol* **14**, 337-345 (2013).
3. Shaffer, A.L. *et al.* A library of gene expression signatures to illuminate normal and pathological lymphoid biology. *Immunol Rev* **210**, 67-85 (2006).
4. Dirmeier, U. *et al.* Latent membrane protein 1 of Epstein-Barr virus coordinately regulates proliferation with control of apoptosis. *Oncogene* **24**, 1711-1717 (2005).
